# Twenty-nine new host records of powdery mildew fungi (Erysiphaceae) in Taiwan with an updated checklist

**DOI:** 10.1186/s40529-025-00471-1

**Published:** 2025-08-25

**Authors:** Yu-Wei Yeh, Meike Piepenbring, Roland Kirschner

**Affiliations:** 1 School of Forestry & Resource Conservation, National Taiwan University, Roosevelt Rd. Sec. 4 No. 1, Taipei City, Taiwan; 2 Mycology Research Group, Faculty of Biological Sciences, Goethe University Frankfurt Am Main, Biologicum, Max-Von-Laue-Straße 13, Frankfurt Am Main, Germany

**Keywords:** Fungal diversity, *Erysiphe*, *Golovinomyces*, ITS sequences

## Abstract

**Background:**

Powdery mildews (Erysiphaceae, Ascomycota) belong to the most important plant pathogenic fungi that cause stress to forest and fruit trees and lead to a considerable loss of economic crops worldwide as well as in Taiwan. The checklist of Erysiphaceae in Taiwan is an important basis to control these pathogens. However, it has been published 25 years ago without any updating, while taxonomic concepts of genera and species changed and many new fungus and host records have been published. We update the checklist of Erysiphaceae by applying new taxonomical concepts and we add several new host records worldwide or for Taiwan based on own, recent collections.

**Results:**

The updated checklist of powdery mildew in Taiwan is presented including data on 13 species of Erysiphaceae on 28 species of host plants. Doubtful records are marked as such and proposed for further investigation. For agricultural application, information on the usage of host plants was added to the checklist. Besides, over twenty specimens of Erysiphaceae on common plants in Taiwan recently collected by the authors were identified based on ITS sequences and anamorphic morphology. Among the collections, twelve represented new host records worldwide, while seventeen were new host records for Taiwan. Six species of Erysiphaceae were found in Taiwan for the first time.

**Conclusions:**

The checklist of Erysiphaceae in Taiwan is an important basis for taxonomic and biogeographical studies as well as for quarantine sanctions referring to powdery mildews. Our results reveal that the host spectra of these pathogens are still incompletely known worldwide. Moreover, the list includes some doubtful old records that might be synonyms or wrong identifications. Morphological characteristics and ITS sequences are usually useful for the identification of Erysiphaceae. Several species in certain genera, however, cannot be distinguished since the variation in sequence data of those closely related species is quite low. New collections and re-investigation of old specimens are imperative to clarify some doubtful records.

## Background

Powdery mildews (Erysiphaceae, Ascomycota) are distributed worldwide and among the economically most important obligate plant pathogenic fungi comprising over 900 species belonging to 18 genera (Shirouzu et al. 2020; Bradshaw et al. 2024a). They can infect over 10,000 species of angiosperms including food crops and ornamental plants (Bradshaw et al 2022a). The traditional classification of Erysiphaceae was based on the teleomorph morphology and host specificity (Braun and Cook 2012). Anamorphic characteristics were largely underestimated since they were either unknown or only partially known in the past.

In tropical/subtropical regions such as Taiwan, the anamorph stage is dominant and can be used for identification only in combination with sequence data since the morphological features of anamorphs are less diagnostic for species identification than features of the teleomorph (Piepenbring et al. 2011; Braun and Cook 2012). Therefore, sequences of the rDNA ITS region have become universal DNA barcodes for species identification of Erysiphaceae (Takamatsu et al. 2015a, b; Meeboon and Takamatsu 2016, 2017a, 2017b, 2020). Multi-gene phylogenetic analyses are used to resolve cryptic species and species complexes, e.g., the *Erysiphe aquilegiae* species complex, the *Erysiphe trifoliorum* species complex, and the *Golovinomyces biocellatus* species complex (Bradshaw et al. 2022b, c, 2023a, b, 2024b, 2025a, 2025b).

The systematic studies of Erysiphaceae in Taiwan began with Sawada (1914) and were mainly contributed by Sawada (1914, 1919, 1930, 1933, 1959) and Yen (1966, 1967) and Yen and Wang (1973) in the early period. In the 1980 s, studies based on morphology and host specificity confirmed several previous records and reported two new host records for Taiwan (Hsieh 1983a, b, c, 1986; Kao and Kuo 1987; Leu and Kuo 1988; Kuo et al. 1989). In 1998, the first comprehensive checklist of Erysiphaceae in Taiwan, which includes both fungal species and their host plants, was compiled by Kuo (1998) including an update of concepts of genera of powdery mildews according to Braun (1987). In the following 15 years, research on powdery mildew in Taiwan mostly focused on prevention and control, with only eight new fungus-host combinations being reported for Taiwan during this period (Huang et al. 2002; Hsieh et al. 2002, 2005; Fu et al. 2004; Cheng et al. 2006; Chen et al. 2008; Kirschner and Chen 2008; Tsay et al. 2011). Meanwhile, more recent new generic concepts of Erysiphaceae were brought up and the most up-to-date monograph of Erysiphaceae was published (Braun and Takamatsu 2000; Braun and Cook 2012). However, several publications of Erysiphaceae in Taiwan were not included in the monograph (Braun and Cook 2012), e.g., the checklist of Kuo (1998) including a record of *Erysiphe izuensis* on *Rhododendron* and of *E. alphitoides* on *Cyclobalanopsis glauca* for Taiwan, *E. liquidambaris* (Leu & Kuo 1988) and the more recent new records for Taiwan of *E. diffusa* on soybean (Chen 2003a), *Oidium murrayae* on the ornamental bush *Murraya paniculata* (Hsieh et al. 2002) and of *Podosphaera xanthii* on the medicinal plant *Physalis angulata* (Cheng et al. 2006). In addition, without re-examination of specimens, Braun & Cook (2012) schematically made new combinations into anamorph genera, which became obsolete by 1 fungus = 1 name nomenclature (Braun 2013). The monograph of Braun & Cook (2012) despite some limitations is a good basis for traditional identifications and the discovery of new hosts. In the past decade, the number of new fungus-host combinations in Taiwan has increased rapidly, with fifty combinations reported (Kirschner and Liu 2014; Kirschner 2015a, b, c; Shen et al. 2015; Liu and Kirschner 2015; Wu and Kirschner 2017; Chen and Kirschner 2018; Yeh et al. 2018; Wang et al. 2019; Lin et al. 2019; Wu et al. 2019; Xiao et al. 2020; Yeh et al. 2020; Yeh et al. 2021; Hsiao et al 2022; Wang et al. 2022; Lin et al. 2022; Chu et al. 2023; Yeh and Kirschner 2024). With numerous records and new taxonomic concepts of Erysiphaceae based on multi-gene phylogenetic analyses, updating the checklist of Erysiphaceae in Taiwan is imperative.

## Methods

### Sampling and morphology

In the context of this study, new host plant specimens with powdery mildews were collected in the wild, botanical gardens as well as from outdoor plantations and potted plants. They were kept in a refrigerator (ca. 8 °C) before processing. For micromorphological study, fresh hyphae and conidia were picked up from the host leaf surface with transparent tape, mounted in 5–10% KOH or purified water and observed at 1000 × magnification. The sizes of conidiophores, foot cells and conidia were measured and noted as mean value ± standard deviation of n measurements with extreme values in brackets. Drawings were made by hand with scaled paper. The specimens were dried by pressing plant organs with fungal infection between paper for few days and subsequent drying on an electrical dryer. Representative specimens were deposited at the National Museum of Natural Science, Taichung, Taiwan (TNM).

### Molecular identification

For molecular identification, fresh conidia and mycelium were collected by a tiny needle for the extraction of genomic DNA. The internal transcribed spacer (ITS) region of ribosomal RNA genes (ITS 1, 5.8S rDNA, ITS2 and bordering short fragments of the 18S and 28S rDNA) was amplified, sequenced, and edited as in Wei and Kirschner (2017). ITS sequences were used for megaBLAST searches at GenBank and deposited in GenBank.

### Data sources for checklist compilation and analysis

Literature research for updating the checklist of powdery mildews in Taiwan was principally based on Kuo (1998) and complemented by additional literature. These publications were retrieved through searches, including Google Scholar, the fungal database of the US Department of Agriculture (Farr and Rossman 2024), and by reviewing the references cited in Kuo (1998) and Braun and Cook (2012). The updated checklist contains information for records of Erysiphaceae fungi and hosts for Taiwan in an excel file (supplementary material 1), including host names, host families, recorded names of fungi, accepted names of fungi, usage of host plants, year of the first record for Taiwan, references, and notes. For the scientific names of hosts and fungi in the checklist, we initially standardized all the species names by consulting databases and monographs and, when necessary, replacing them with the accepted species names, except for recorded names of fungi, which remained original to trace historical records. This ensured that any discrepancies between the records reflected true differences in taxa rather than simply nomenclatural variations. TROPICOS (www.tropicos.org) and International Plant Names Index were utilized for correcting scientific names of host plants, host families and synonyms. Fungal names were standardized by Index Fungorum (www.indexfungorum.org) and Braun and Cook (2012), and fungal names with uncertain identification were marked with question marks as doubtful records. For those fungal species that cannot be separated by ITS sequences (e.g., *Erysiphe aquilegiae* species complex), host-based species names are adopted following Braun and Cook (2012), if available, e.g., *E*. *begoniae*, *E*. *pileae*, and *E*. *sedi*. The authors of the scientific names were taken from above-mentioned three databases. Records with invalid names or not identified as species were included in the checklist because they most probably refer to distinct species and host records, whereas synonyms are not included. The usage of host plants was mainly based on Chang-Yang et al. (2022). The references cited by Kuo (1998) are not listed in the checklist, but references for missing records and works published after 1998 are included. The statistical analysis and charts were performed using R version 4.0.2 (R Core Team 2020) with RStudio base functions. For the analyses, species and infraspecific taxa were considered, while records limited to genus level or those with incomplete identification in the literature (e.g., *Oidium* sp. and *Erysiphe* aff. *betae*) were excluded.

## Results

In the context of this study, more than forty specimens of Erysiphaceae in the anamorphic stage were collected in Taiwan during the last 8 years. They represent 13 distinct species (including species complexes) on 28 different host plant species. Twenty-nine of these plant species were found to be the new hosts of powdery mildews in Taiwan or worldwide. Twelve collections of Erysiphaceae were found on new host species worldwide, namely *Erysiphe aquilegiae* species complex on *Nicotiana plumbaginifolia*, *Erysiphe diffusa* on *Hibiscus sabdariffa*, *Erysiphe elevata* on *Radermachera sinica*, *Erysiphe hommae* on *Scutellaria barbata*, *Erysiphe necator* on *Causonis japonica*, *Erysiphe trifoliorum* species complex on *Myriophyllum aquaticum*, *Golovinomyces magnicellulatus* on *Nicotiana plumbaginifolia*, *Podosphaera xanthii* on *Ixeris chinensis*, *Mikania micrantha*, *Parthenium hysterophorus*, *Scoparia dulcis*, and *Strobilanthes cusia*. Seventeen collections are new host records for Taiwan, *Erysiphe begoniae* on *Begonia* sp., *Erysiphe elevata* on *Tabernaemontana divaricata*, *Erysiphe necator* on *Ampelopsis brevipedunculata*, *Erysiphe nyctaginacearum* on *Mirabilis jalapa*, *Erysiphe pileae* on *Pilea microphylla* and *Pellionia scabra*, *Erysiphe sedi* on *Kalanchoe blossfeldiana, K. delagoensis*, *K. laetivirens*, and *K. thyrsiflora*, *Erysiphe syringae* on *Ligustrum liukiuense*, *Erysiphe trifoliorum* species complex on *Melilotus officinalis* and *Podosphaera xanthii* on *Acmella uliginosa*, *Cleome rutidosperma*, *Clinopodium gracile*, *Cyanthillium cinereum*, and *Verbena* sp. Among these records, six species of Erysiphaceae were found for the first time in Taiwan, namely *Erysiphe begoniae*, *Erysiphe hommae*, *Erysiphe nyctaginacearum*, *Erysiphe pileae*, *Erysiphe syringae*, and *Golovinomyces magnicellulatus*. The taxonomy of powdery mildews in this study was based on anamorph morphology, host specificity, and molecular identification with BLAST analysis. The specimens of new records of species of Erysiphaceae for Taiwan are briefly characterized below according to alphabetically arranged fungal names. Minor differences in presentation style result from the distinct methods of each contributing author and the conditions of the specimens.

An updated checklist of Erysiphaceae in Taiwan based on the list from Kuo (1998) was compiled and uploaded to Zenodo in the Excel file format (10.5281/zenodo.15294196). The checklist will be converted to the GBIF (Global Biodiversity Information Facility) format and uploaded to the GBIF database to enhance the standardization and reusability of the data. According to the checklist, there are currently around 109 species of powdery mildew known for Taiwan, affecting approximately 246 host species across 75 host families excluding doubtful records. The checklist contains the updated scientific names of hosts and fungi and provides information for further application, e.g., host family, and usage of host plants. Kuo’s (1998) checklist was carefully compiled and largely complete, with only a few omissions including published records overlooked by the same author (Kuo et al. 1991). Most of these omissions resulted from poor information circulation at the time, such as records published in a Japanese local journal (Tanda and Su 1995). The records missing from Kuo’s list (1998) and those reported after 1998 were revised and included. Besides, by statistical analysis the changes in the number of powdery mildew species and their hosts over time are investigated (see Discussion Section). The references cited by Kuo (1998) are not repeated here, but references for missing records and those published after 1998 are provided.

**Fig. 1:**
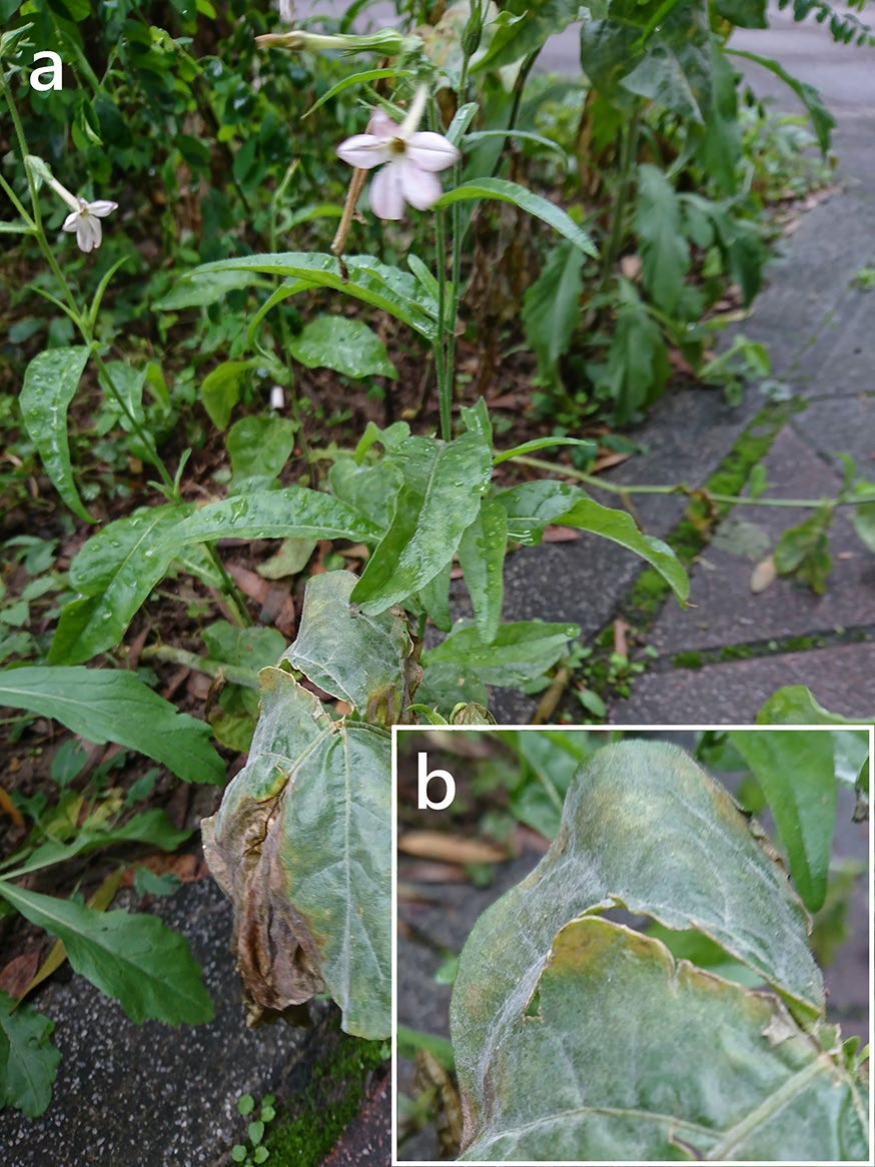
*Erysiphe aquilegiae* species complex on *Nicotiana plumbaginifolia* (AKII 0127). **a**. Powdery mildews symptoms on leaves. **b**. Magnified view of the infected leaf surface

***Erysiphe aquilegiae*** species complex on *Nicotiana plumbaginifolia* (Solanaceae) Figure 1

**Specimens** On *Nicotiana plumbaginifolia* Viv., TAIWAN, Taipei City, Wenshan District, Chin-Nan Temple, ca. 24.980099 N, 121.586507 E, ca. 200 m a.s.l., 4. Apr. 2021, AKII 0127 (TNM), ITS sequence in GenBank PV056439; New Taipei City, Zhonghe District, Xiulang Bridge, ca. 24.990035 N, 121.527755 E, ca. 5 m a.s.l., 24. Jan. 2024, AKII 0194 (TNM), ITS sequence in GenBank PV056459.

**Notes** When ITS sequences exceeding 600 bp were compared, there was 99% to 100% identity with 0 to 1 different bp between our specimens and published specimens labeled as *E. aquilegiae* or other species supposed to belong to the *Erysiphe aquilegiae* species complex in GenBank, e.g., *Pseudoidium hortensiae* and *Erysiphe begoniae*. The fungi were identified as *Erysiphe aquilegiae* species complex since only rDNA sequences are insufficient to unravel the species in the *Erysiphe aquilegiae* species complex (Bradshaw et al. 2024b). *Nicotiana plumbaginifolia* has not yet been recorded as host species of species of the *Erysiphe aquilegiae* species complex worldwide (Braun and Cook 2012; Farr and Rossman 2024). Therefore, we consider these specimens to be the first record of *Erysiphe aquilegiae* species complex on *Nicotiana plumbaginifolia*.

***Erysiphe begoniae*** R.Y. Zheng & G.Q. Chen on *Begonia* sp. (Begoniaceae).

**Specimen** On *Begonia* sp., TAIWAN, Nantou County, Lugu Township, National Taiwan University Experimental Forest, Fenghuang Nature Education Area, ca. 23.728710 N, 120.787910 E, ca. 860 m a.s.l., 22. Apr. 2023, AKII 0171 (TNM), ITS sequence in GenBank PV056447.

**Notes** The ITS sequence was 99% to 100% identical to over twenty published sequences exceeding 600 bp of *E. aquilegiae* or other species supposed to be *Erysiphe aquilegiae* species complex in GenBank, whereas the fungus was identified as *Erysiphe begoniae* based on host specificity (Braun and Cook 2012). Presently, it is not possible to distinguish species in the *Erysiphe aquilegiae* species complex only based on rDNA sequences (Bradshaw et al. 2024b). Although three species—*Erysiphe begoniae*, *Erysiphe begoniicola*, and *Golovinomyces orontii*—have been recorded on *Begonia* spp. in several countries, they can still be distinguished by their morphological characteristics (Braun and Cook 2012; Farr and Rossman 2024). The record by Sawada (1959) was published under the name *Oidium ellipticum* which is invalid due to the lack of Latin description, and needs to be re-examined. We, therefore, treat this specimen as the host record of *E. begoniae* on *Begonia* sp. in Taiwan.

**Fig. 2:**
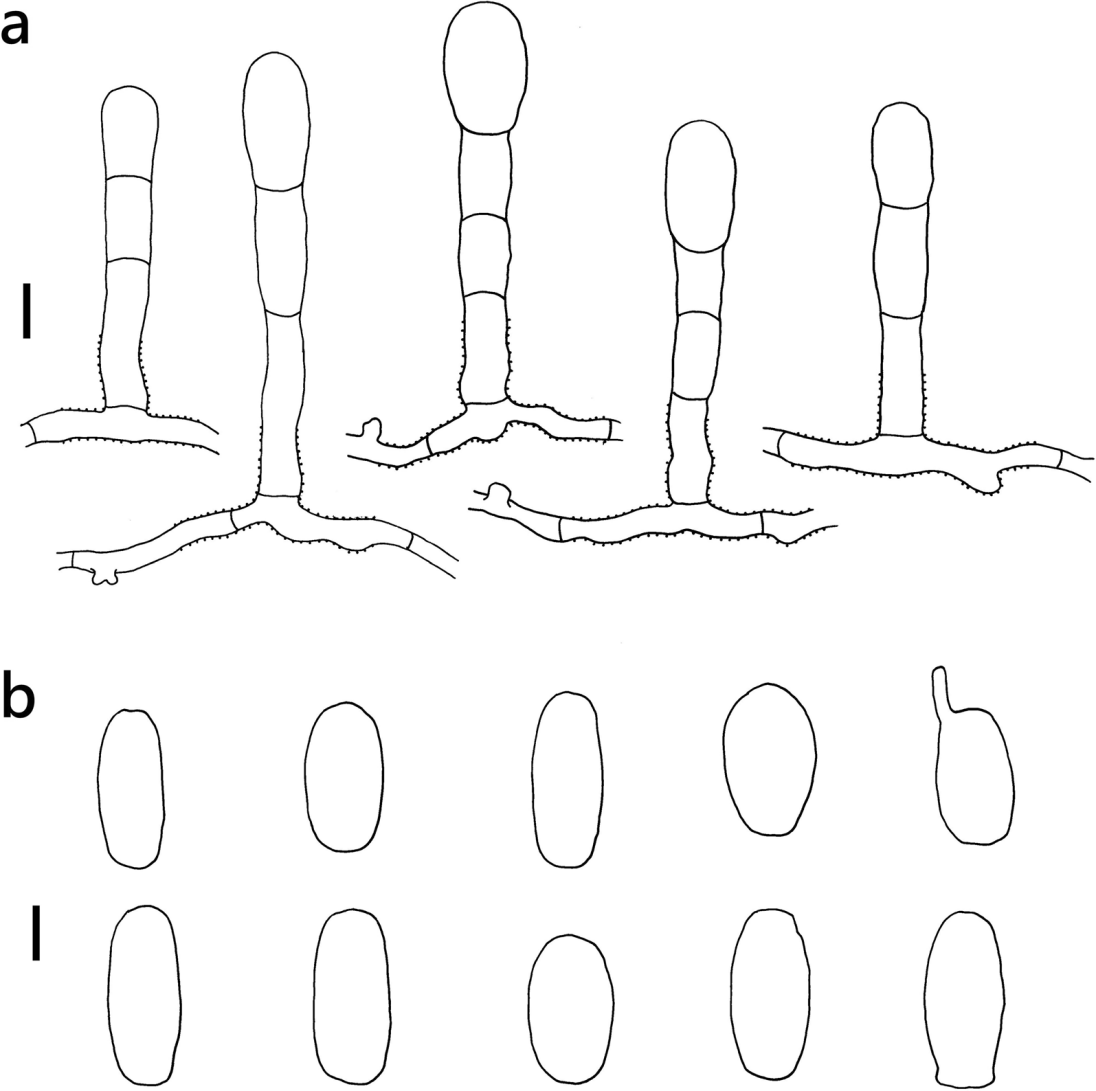
*Erysiphe diffusa* on *Hibiscus sabdariffa* (R. Kirschner 5084). **a**. Hyphae with nipple-shaped appressoria and conidiophores with curved foot cells. **b**. Conidia. Scale bars = 10 μm

***Erysiphe diffusa*** (Cooke & Peck) U. Braun & S. Takam. on *Hibiscus sabdariffa* (Malvaceae) Figure 2

**Description** Mycelium amphigenous on leaves and petioles, forming irregular patches. Hyphae hyaline, smooth, 4–6 μm wide, with solitary, nipple-shaped appressoria. Conidiophores terminal on the surface of mother cells, mostly composed of 3 cells, (36–)42–61(–77) × 7–9(–10) μm (n = 30), foot cells slightly curved, (20–)24–37(–49) × 7–9(–10) μm (n = 30), basal septum of the foot cell mostly at almost the same level as surface of supporting hypha. Conidia solitary, ellipsoid to doliiform without fibrosin bodies, (23–)27–32(–34) × (13–)14–16(–18) μm (n = 30). Germination subterminal, conidial appressoria not found.

**Specimen** On *Hibiscus sabdariffa* L., TAIWAN, Taipei City, Daan District, Jinan Market, ca. 25.028472 N, 121.529265 E, ca. 10 m a.s.l., 12. Jan. 2021, R. Kirschner 5084 (TNM), ITS sequence in GenBank PV056471.

**Notes** When ITS sequences exceeding 630 bp were compared, our specimen was 100% identical to over twenty published specimens labeled as *E. diffusa* in GenBank, whereas the identity with other species was 98% or lower, with more than 7 bp difference. The morphological characteristics of our specimen are mostly the same as those provided by Braun and Cook (2012). *Hibiscus sabdariffa* has not yet been recorded as the host of *Erysiphe diffusa* worldwide (Braun and Cook 2012; Farr and Rossman 2024).

**Fig. 3:**
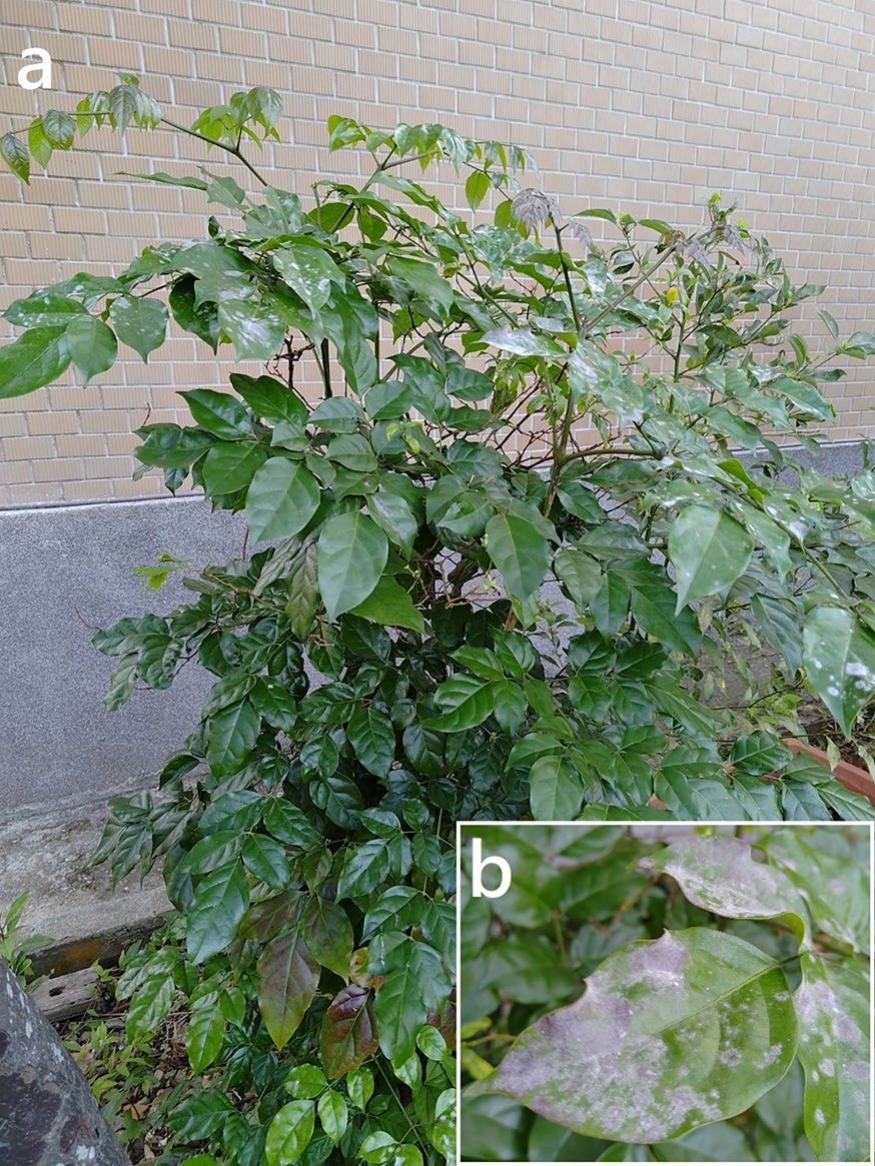
*Erysiphe elevata* on *Radermachera sinica* (R. Kirschner 6121). **a**. Powdery mildews symptoms on leaves. **b**. Magnified view of the infected leaf surface

***Erysiphe elevata*** (Burrill) U. Braun & S. Takam. on *Radermachera sinica* (Bignoniaceae) Figure 3

**Specimen** On *Radermachera sinica* (Hance) Hemsl., TAIWAN, Taipei City, Daan District, National Taiwan University, side entry of Forestry Department, ca. 25.016881 N, 121.538634 E, ca. 15 m a.s.l., 24. Mar. 2025, R. Kirschner 6121 (TNM), ITS sequence in GenBank PV422615.

**Notes** The ITS sequence was 100% identical to over twenty published specimens labeled as *E. elevata* or *E. vaccinii* f. *elevata* from Apocynaceae and Bignoniaceae in GenBank. In BLAST searches, these sequences differed from those on *Vaccinium* hosts by a transition from C to T at the same position near the 3’ end. In contrast, Bradshaw et al. (2025a) stated that the sequences were not different and reduced *E. elevata* to a forma of *E. vaccinii* Schwein. We treat this fungus as *E. elevata* and not as forma of *E. vaccinii*, since the phylogenetic tree of *E. vaccinii* s. lat. demonstrated that its subclades correspond to specific hosts (Bradshaw et al. 2025a). Furthermore, the anamorph of the taxa on *Vaccinium* hosts (Ericaceae) is rare or absent, but abundant in *E. elevata* on Bignoniaceae (Tymon et al. 2022). *Radermachera sinica* has not yet been recorded as the host of *Erysiphe elevata* worldwide (Braun and Cook 2012; Farr and Rossman 2024).

**Fig. 4:**
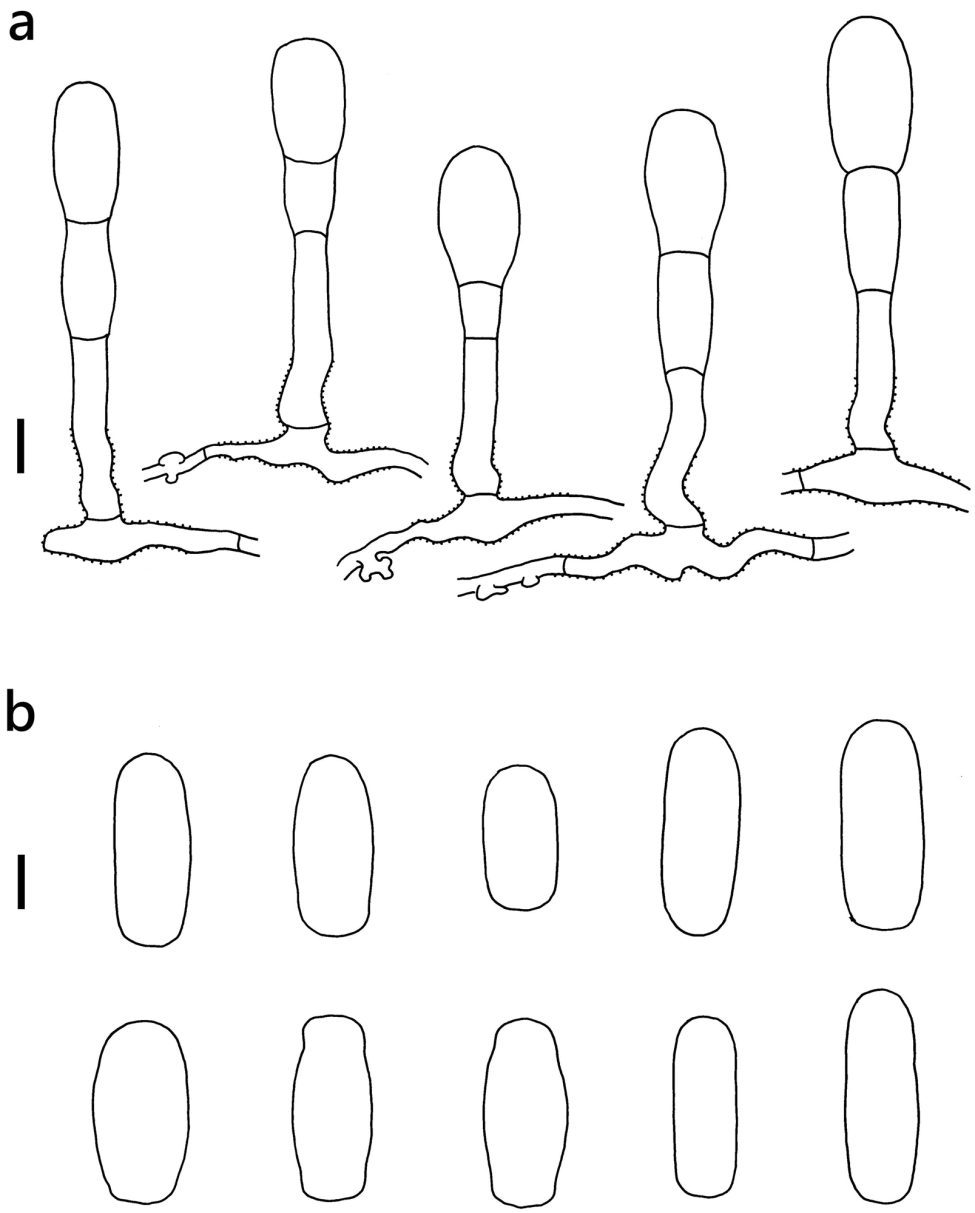
*Erysiphe elevata* on *Tabernaemontana divaricata* (AKII 0147). **a**. Hyphae with nipple-shaped or lobed appressoria and conidiophores with curved foot cells. **b**. Conidia. Scale bars = 10 μm

***Erysiphe elevata*** (Burrill) U. Braun & S. Takam. on *Tabernaemontana divaricata* (Apocynaceae) Figure 4

**Description** Mycelium amphigenous on leaves, forming irregular patches. Hyphae hyaline, walls thin, smooth, 3–5 μm wide. Appressoria lobed or nipple-shaped, solitary or in opposite pairs. Conidiophores terminal on the surface of mother cells, mostly composed of 3 cells, (40–)49–66(–83) × 6–8 μm (n = 30), foot cells mostly curved, constricted at basal septum, (29–)33–42(–47) × 6–8 μm (n = 30), basal septum of the foot cell mostly at almost the same level as surface of supporting hypha. Conidia solitary, cylindrical to doliiform, without fibrosin bodies, (27–)29–37(–40) × (12–)13–16(–18) μm (n = 30). Germination not found.

**Specimen** On *Tabernaemontana divaricata* (L.) R. Br. ex Roem. & Schult., TAIWAN, Taitung County, Taitung City, Shanghai Street, ca. 22.761237 N, 121.145192 E, ca. 15 m a.s.l., 16. Feb. 2022, AKII 0147 (TNM) = R. Kirschner 5440 (TNM), ITS sequence in GenBank PV056445.

**Notes** The ITS sequence was 100% identical to over twenty published specimens labeled as *E. elevata* or *E. vaccinii* f. *elevata* in GenBank, whereas the fungus was identified as *E. elevata* since the anamorphic characteristics of our specimens were mostly identical to the description of *E. elevata* given by Braun and Cook (2012), except for the length of conidiophores. This fungus-host combination was hitherto recorded for China (Xu et al. 2021; Farr and Rossman 2024). We, therefore, treat this specimen as the first record of *E. elevata* on *Tabernaemontana divaricata* in Taiwan*.* For the species status see above under *Radermachera sinica*. Bradshaw et al. (2025a) suggested that the records of *E. elevata* on Apocynaceae may reveal a separate taxon in future multigene analyses.

**Fig. 5:**
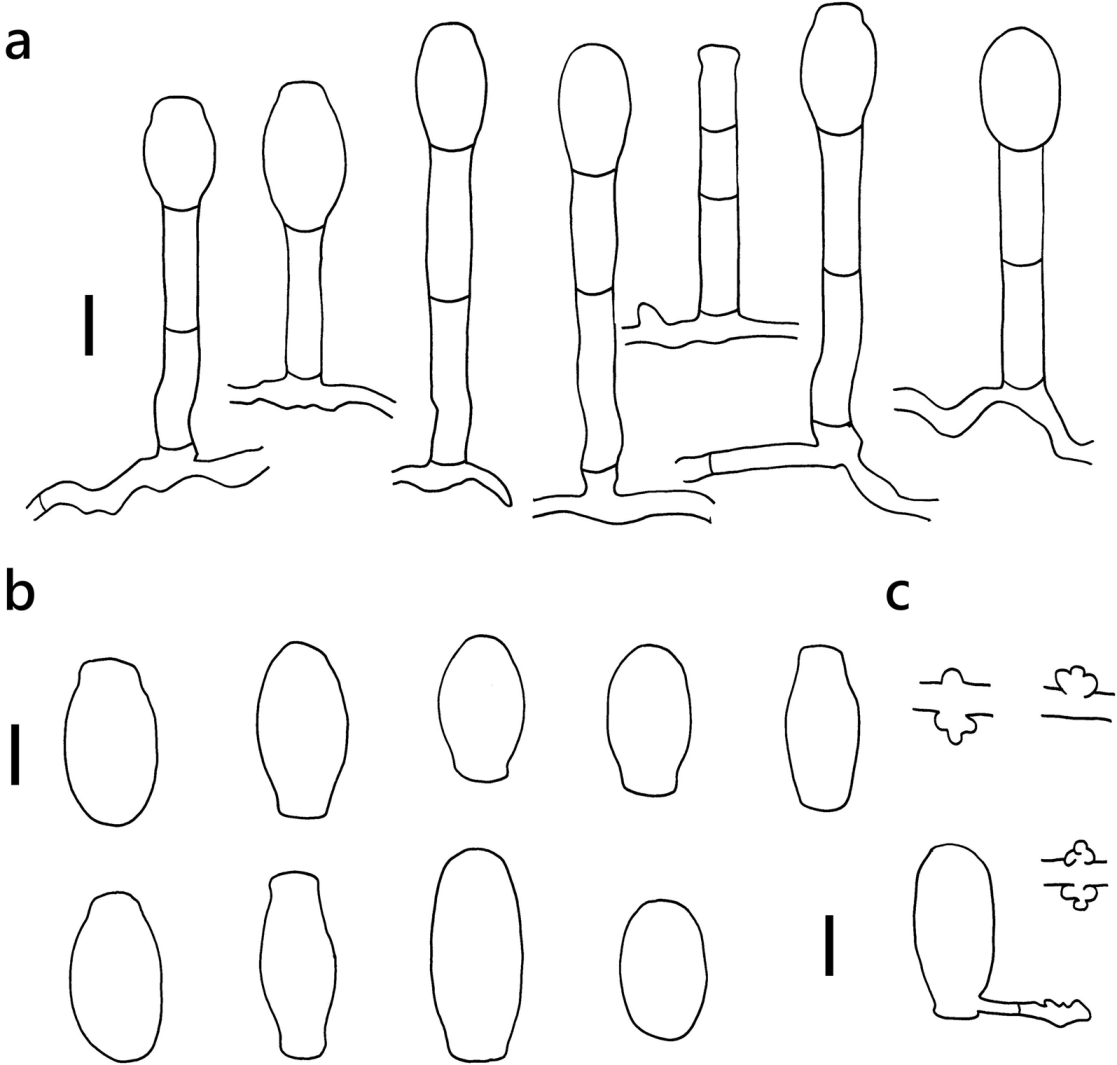
*Erysiphe hommae* on *Scutellaria barbata* (AKII 0131). **a**. Hyphae with lobed appressoria and conidiophores with slightly curved foot cells. **b**. Conidia. **c**. Conidia with germination hypha and appressoria. Scale bars = 10 μm

***Erysiphe hommae*** U. Braun on *Scutellaria barbata* (Lamiaceae) Figure 5

**Description (based on AKII 0131)** Mycelium amphigenous on leaves and petioles, forming irregular patches. Hyphae hyaline, smooth, 4–5 μm wide. Appressoria lobed or nipple-shaped, solitary or in opposite pairs. Conidiophores terminal on the surface of mother cells, mostly composed of 3 cells, (30–)47–72(–83) × (6–)7–8(–10) μm (n = 30), foot cells straight or slightly curved, (25–)26–37(–55) × (5–)6–8(–10) μm (n = 30), basal septum of the foot cell mostly at almost the same level as surface of supporting hypha. Conidia solitary, cylindrical to doliiform without fibrosin bodies, (20–)26–35(–42) × (12–)13–16(–17) μm (n = 30). Germination subterminal or lateral with lobed appressorium.

**Specimens** On *Scutellaria barbata* D. Don, TAIWAN, Taipei City, Zhongzheng District, Taipei Botanical Garden, ca. 25.031880 N, 121.509477 E, ca. 10 m a.s.l., 23. Apr. 2021, AKII 0131 (TNM); same collection site, 7. Apr. 2023, AKII 0169 (TNM), ITS sequence in GenBank PV056448.

**Notes** When ITS sequences exceeding 630 bp were compared, our specimen was 99% identical to published specimens labeled as *E. aquilegiae* or other species supposed to belong to the *Erysiphe aquilegiae* species complex in GenBank, whereas the fungus was classified as *E. hommae* based on host specificity (Braun and Cook 2012). Besides, the morphological features of our specimen mostly match the description of *E. hommae* by Braun and Cook (2012), except for the length of conidiophores. However, *E. hommae* cannot be separated from the *Erysiphe aquilegiae* species complex only based on rDNA sequence, which needs further study and additional DNA markers to resolve this complex (Bradshaw et al. 2024b). *S. barbata* has not yet been recorded as the host of *E. hommae* worldwide (Braun and Cook 2012; Farr and Rossman 2024). There are two records of powdery mildew on the other species of *Scutellaria* in Taiwan, *E. galeopsidis* on *S. formosana*, and *Oidium ellipticum* on *S. playfairi* (Amano 1986; Sawada 1959). The record published by Amano (1986) is considered invalid due to the lack of evidence, and the fungus name of the record by Sawada (1959) is invalid due to the lack of Latin description.

**Fig. 6:**
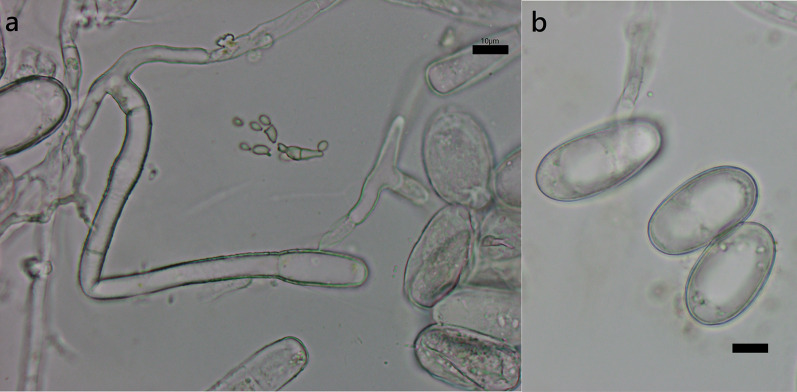
*Erysiphe necator* on *Ampelopsis brevipedunculata* (R. Kirschner 6008). **a**. Hyphae with lobed appressorium and conidiophore with slightly curved foot cell. **b**. Conidia. Scale bars = 10 μm

***Erysiphe necator*** Schwein. on *Ampelopsis brevipedunculata* (Vitaceae) Figure 6

**Specimen** On *Ampelopsis brevipedunculata* (Maxim.) Trautv., TAIWAN, New Taipei City, Yonghe District, Fuhe Bridge, ca. 25.000619 N, 121.527293 E, ca. 10 m a.s.l., 21. Apr. 2024, R. Kirschner 6008 (TNM), ITS sequence in GenBank PV056469.

**Notes** When comparing BLAST matches exceeding 600 bp, there was 99% to 100% identity with 0 to 2 bp different bp between our specimen and published specimens labeled as *E. necator* in GenBank, whereas the identity with other species was 94% or lower, with more than 30 different bp. *A. brevipedunculata* has not yet been recorded as the host of *E. necator*, but *E. necator* on *A. brevipedunculata* var. *heterophylla* [= *A. glandulosa* var. *heterophylla*] has been reported (Braun and Cook 2012; Nomura et al. 2003). This specimen, therefore, is supposed to be the first host record of *E. necator* on *A. brevipedunculata* in Taiwan.

**Fig. 7:**
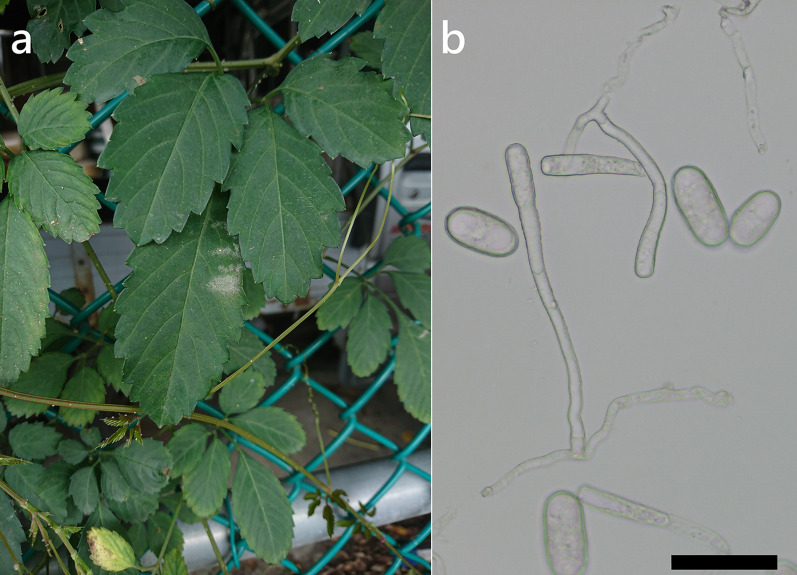
*Erysiphe necator* on *Causonis japonica* (R. Kirschner 5997). **a**. Powdery mildew symptoms on leaves. **b**. Conidia and conidiophores with slightly curved foot cells. Scale bar = 50 μm

***Erysiphe necator*** Schwein. on *Causonis japonica* (Vitaceae) Figure 7

**Description** Mycelium amphigenous, white. Hyphae hyaline, smooth to verruculose, 3–5 µm wide. Appressoria lobed, solitary, in single opposite pairs, or in two neighboring opposite pairs. Conidiophores composed of foot cell and up to 2 cells being shorter than foot cell, verruculose, including terminal conidium initial (43–)80–135(–156) µm long (n = 20). Foot cell curved to sinuous or straight, cylindrical, basal septum 0–5 µm above upper surface of hyphal mother cell, (12–)35–68(–80) × 6–8(–10) µm (n = 20). Conidia solitary, ellipsoidal, oblong-cylindrical, or slightly clavate, with broadly rounded ends, smooth, (32–)39–44(–46) × (17–)17.5–20 µm (n = 20), germinating at one or two ends with long hyphae or sessile lobed appressoria.

**Specimens** On *Causonis japonica* (Thunb.) Raf. [= *Cayratia japonica* (Thunb.) Gagnep.], TAIWAN, New Taipei City, Zhonghe District, Huanhe East Rd. Sec. 4, ca. 24.995869 N, 121.527730 E, ca. 10 m a.s.l., 5. Apr. 2024, R. Kirschner 5997 (TNM), ITS sequence in GenBank PV056470; ibid., 19. May 2024, R. Kirschner 5997-C (TNM).

**Notes** The ITS sequence was 99% to 100% identical to over thirty published ones exceeding 600 bp of *E. necator* with 0 to 1 bp difference in GenBank, whereas the identity with other species was 94% or lower, with more than 30 different bp. This fungus-host combination was recorded hitherto only for Japan with the host being *Cayratia japonica*, though it is not considered valid due to the lack of evidence (Amano 1986; Farr and Rossman 2024). We, therefore, consider these specimens to be the first record of *Erysiphe necator* on *Causonis japonica* worldwide.

***Erysiphe nyctaginacearum*** (Hosag.) Meeboon & S. Takam. on *Mirabilis jalapa* (Nyctaginaceae).

**Specimens** On *Mirabilis jalapa* L., TAIWAN, Taipei City, Wanhua District, Lung-Shan Elementary School, ca. 25.035771 N, 121.496208 E, ca. 10 m a.s.l., 12. Mar. 2021, AKII 0122, ITS sequence in GenBank PV056441; Nantou County, Lugu Township, National Taiwan University Experimental Forest, Fenghuang Nature Education Area, ca. 23.729412 N, 120.790430 E, ca. 800 m a.s.l., 07. Feb. 2020, R. Kirschner 4874 (TNM), ITS sequence in GenBank PV056467; ibid., 21. Nov. 2020, R. Kirschner 5069 (TNM), ITS sequence in GenBank PV056453.

**Notes** When ITS sequences exceeding 630 bp were compared, our specimen was 99% identical to published specimens labeled as *E. trifoliorum* or other species supposed to belong to the *Erysiphe trifoliorum* species complex in GenBank. The fungus was identified as *E. nyctaginacearum* by the host range. This fungus-host combination was recorded hitherto only for India and Thailand (Farr and Rossman 2024; Meeboon and Takamatsu 2017b). Currently, rDNA sequences are insufficient to distinguish *E. nyctaginacearum* from this complex (Bradshaw et al. 2025a).

**Fig. 8:**
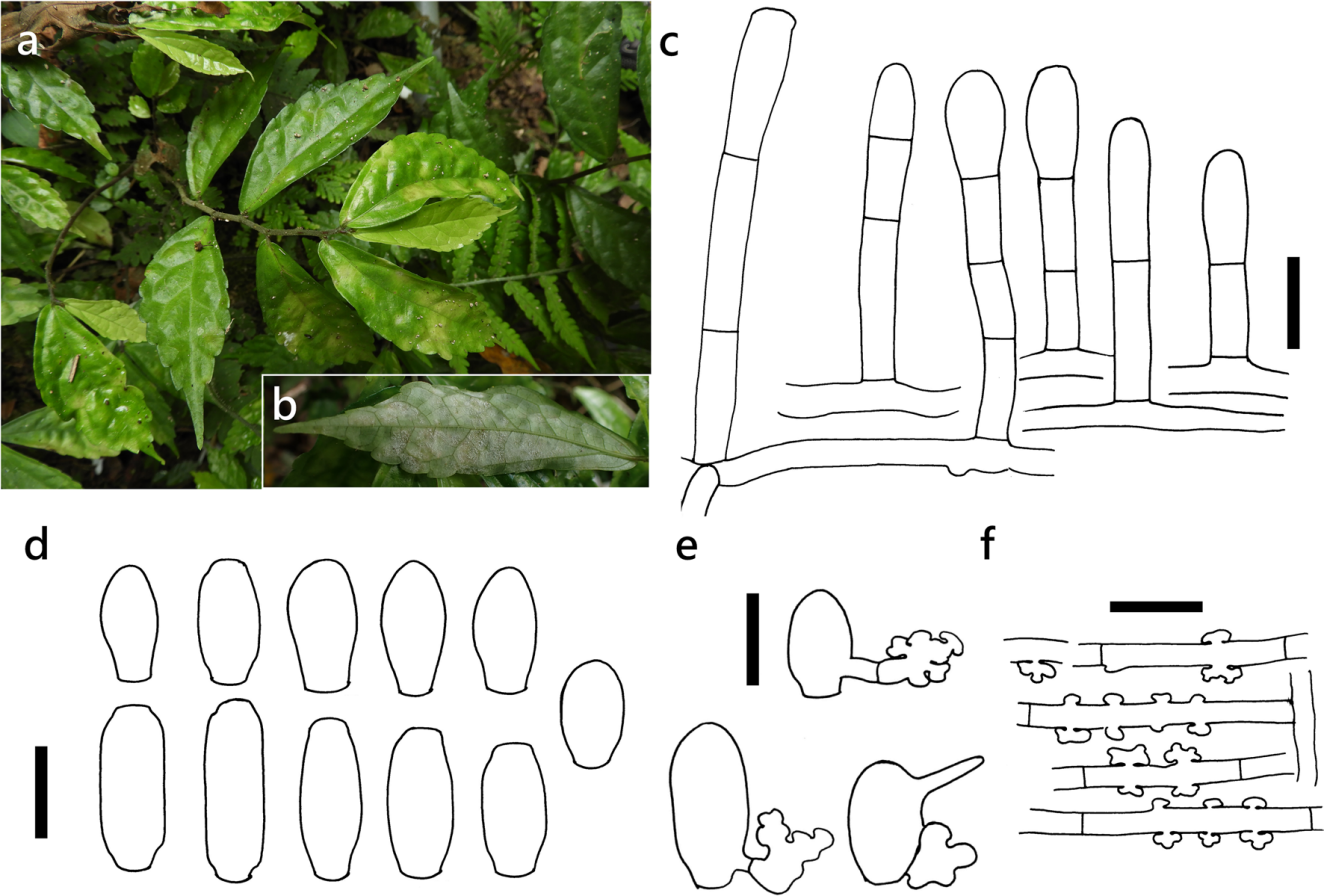
*Erysiphe pileae* on *Pellionia scabra* (R. Kirschner 4930). **a**. Powdery mildews symptoms on leaves. **b**. Magnified view of the infected leaf surface. **c**. conidiophores. **d**. Conidia. **e**. Conidia with germination hyphae and appressoria. **f**. Lobed and nipple-shaped appressoria. Scale bars = 20 μm

***Erysiphe pileae*** U. Braun on *Pellionia scabra* (Urticaceae) Figure 8

**Description** Colonies predominantly hypophyllous, exceptionally also epiphyllous, associated with pale green to green-yellowish discolored leaf areas. Hyphae verruculose, 3–7 μm wide. Hyphal appressoria nipple-shaped and single, or lobed to strongly lobed, and then rarely single, but mostly in opposite pairs, which are often aligned at a row of up to four along the same hyphal cell. Conidiophores arising singly from middle of hyphal mother cell, composed of foot cell and up to two further cells, verruculose, uppermost cell with fine longitudinal striation, (38–)49–71(–80) × (7–)7.5–9(–10) μm (n = 30). Foot cell straight, cylindrical, lower septum almost at the same level as upper surface as mother hypha, (14–)21–32(–36) × (6–)7–8(–9) μm (n = 30). Conidia solitary, verruculose, primary conidia broadly clavate or ellipsoid with broadly rounded or slightly narrowed apex, (24–)25–30(–31) × (12–)12–15(–16) μm (n = 30), secondary conidia cylindrical, broadly cylindrical or doliiform with truncate ends, 28–40 × (11–)13–15(–16) μm (n = 30), germination at the base or apex with short germination hypha terminating in a strongly lobed appressorium.

**Specimen** On *Pellionia scabra* Benth., New Taipei City, Tucheng District, Tung Blossom Park, ca. 24.149033 N, 121.449723 E, below 400 m a.s.l., 03. May 2020, R. Kirschner 4930 (TNM), ITS sequence in GenBank PV125001.

**Notes** The ITS sequence was 100% identical with other sequences of the *E. aquilegiae* clade, including *E. pileae* (LC010059) which also occurs on Urticaceae. On the same host species, Sawada (1959) found a powdery mildew fungus which was labeled with the invalid name *Oidium oblongisporum* Sawada and which is considered conspecific with the fungus on *Tamarindus indica*. Sawada’s specimen on *P. scabra* differed by its mainly epiphyllous colonies, longer conidia (32–54 μm), and shorter conidiophores (24–28 μm). In spite of the close area and same host, the description of *O. oblongisporum* rather excludes than supports conspecificity with our newly collected material. Other records of powdery mildew species on *Pellionia* hosts are not known. Our reason for choosing *E. pileae* for our specimen on *P. scabra* is that *E. pileae* is considered to be specific for Urticaceae (Braun & Cook 2012). The size range of conidia given for *E. pileae* by Zheng & Chen (1981) of 23–33 × 13–15 μm is almost identical to that found in our specimen.

The nomenclature of *E. pileae* suffered from confusion which was resolved in Index Fungorum (www.indexfungorum.org). The species name was originally published invalidly independently by Sawada in 1951 and Bunkina in 1974 (Braun 1981). Two independent attempts to validate the names from Sawada and Bunkina were done by Zheng & Chen (1981) and Braun (1981), respectively. The publication by Braun was from September, but by Zheng & Chen from December of the same year. Thus, the slightly earlier published name is the valid one (Index Fungorum).

**Fig. 9:**
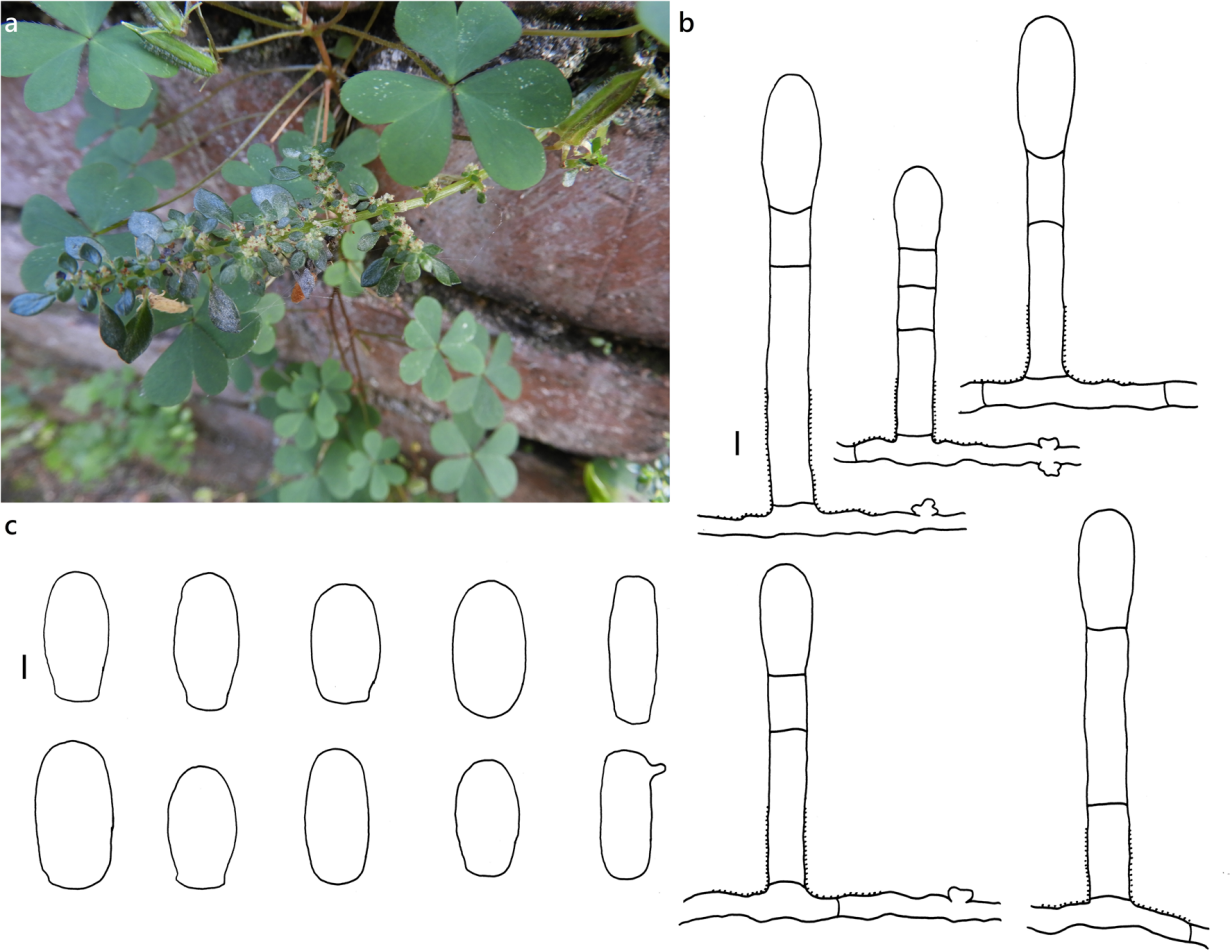
*Erysiphe pileae* on *Pilea microphylla* (AKII 0007). **a**. Powdery mildew symptoms on leaves. **b**. Hyphae with lobed appressoria and conidiophores. **c**. Conidia. Scale bars = 10 μm

***Erysiphe pileae*** U. Braun on *Pilea microphylla* (Urticaceae) Figure 9

**Description (based on AKII 0007 and AKII 0010)** Mycelium amphigenous on leaves, forming irregular patches. Hyphae hyaline, walls thin, smooth, 4–6 μm wide. Appressoria lobed or nipple-shaped, solitary or in opposite pairs. Conidiophores terminal on the surface of mother cells, mostly composed of 3 cells, 45–100 μm, foot cells straight, (21–)28–48(–57) × (7–)8–10 μm (n = 25), basal septum of the foot cell mostly at almost the same level as surface of supporting hypha. Conidia ellipsoid-ovoid to doliiform without fibrosin bodies, (22–)26–30(–33) × (14–)15–17(–18) μm (n = 30). Germ tube lateral.

**Specimens** On *Pilea microphylla* (L.) Liebm., TAIWAN, Taoyuan City, Daxi District, Daxi Old Street, ca. 24.885510 N, 121.287712 E, ca. 100 m a.s.l., 12. Aug. 2018, AKII 0007 (= R. Kirschner 4713) (TNM); New Taipei City, Shenkeng District, Shenkeng Old Street, ca. 25.001701 N, 121.614737 E, ca. 30 m a.s.l., 7. Apr. 2019, AKII 0010, ITS sequence in GenBank PV056438; Taipei City, Daan District, Changxing Street, ca. 25.015797 N, 121.546269 E, ca. 15 m a.s.l., 27. Jan. 2020, R. Kirschner 4873, ITS sequence in GenBank PV056468; Taipei City, Wenshan District, Chinan Temple, ca. 24.980018 N, 121.586636 E, ca. 200 m a.s.l., 9. Aug. 2020, R. Kirschner 4985 (TNM).

**Notes** When ITS sequences exceeding 630 bp were compared, our specimens were 99% to 100% identical to sequences of the *E. aquilegiae* complex, including *E. pileae* which also occurs on Urticaceae. Our specimens were identified as *E. pileae* based on the host specificity, as Braun and Cook (2012) outlined. The anamorphic features they described for *E. pileae* mostly match our specimens, except for the length of conidiophores. Multilocus analyses are necessary to clarify the phylogenetic and taxonomic status of *E. pileae*. Hitherto, no record of powdery mildews exists on *Pilea microphylla* in Taiwan (Kuo 1998; Farr and Rossman 2024). This specimen is the first record of *E. pileae* on *Pilea microphylla* in Taiwan.

**Fig. 10:**
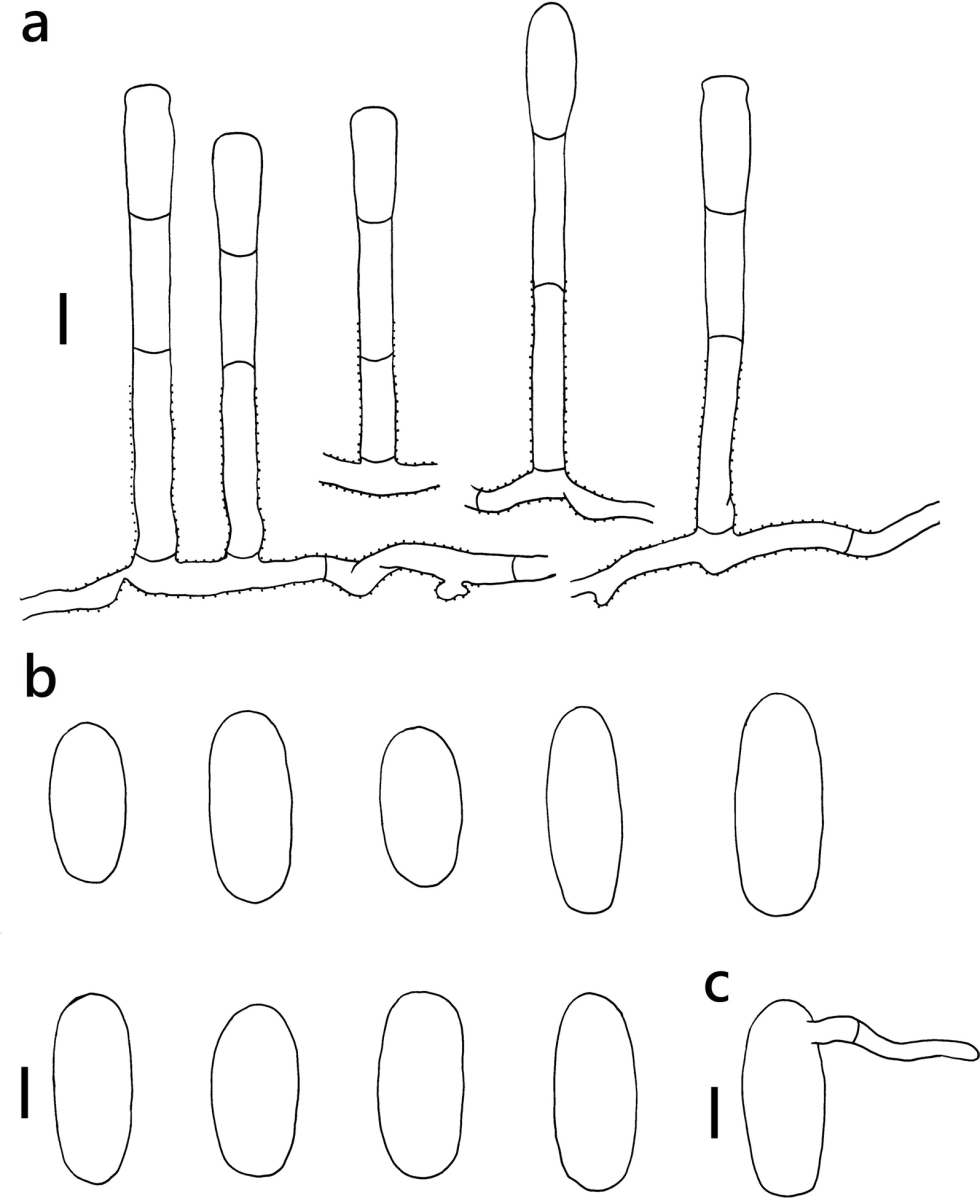
*Erysiphe trifoliorum* species complex on *Melilotus officinalis* (R. Kirschner 4931). **a**. Hyphae with lobed appressoria and conidiophores with slightly curved foot cells. **b**. Conidia. **c**. Conidium with germination hypha. Scale bars = 10 μm

***Erysiphe trifoliorum*** species complex on *Melilotus officinalis* (Fabaceae) Figure 10

**Description (based on R. Kirschner 4931)** Mycelium amphigenous on leaves, forming irregular patches. Hyphae hyaline, walls thin, smooth, 4–7 μm wide. Appressoria lobed or nipple-shaped, solitary. Conidiophores terminal on the surface of mother cells, mostly composed of 3 cells, (47–)62–80(–87) × (5–)6–7 μm (n = 30), foot cells straight or slightly curved, (32–)39–52(–60) × (5–)6–7 μm (n = 30), basal septum of the foot cell mostly at almost the same level as surface of supporting hypha. Conidia solitary, ellipsoid-ovoid to doliiform without fibrosin body, (31–)35–42(–45) × (14–)16–18(–19) μm (n = 30). Germination simple.

**Specimen** On *Melilotus officinalis* (L.) Lam., TAIWAN, New Taipei City, Bali District, Wazihwei Beach, ca. 25.166548 N, 121.410307 E, ca. 5 m a.s.l., 22. Mar. 2020, AKII 0063, ITS sequence in GenBank PV056442; Taipei City, Daan District, National Taiwan University, in front of Chemical Engineering Department, ca. 25.018380 N, 121.538330 E, ca. 10 m a.s.l., 2. May. 2020, R. Kirschner 4931 (TNM), ITS sequence in GenBank PV422609.

**Notes** When ITS sequences exceeding 600 bp were compared, our specimen was 99% to 100% identical to published specimens labeled as *E. pisi* or *Erysiphe trifoliorum* in GenBank. We first chose *E. pisi* for our specimens on *M. officinalis* based on the host range of *E. pisi* (Braun and Cook 2012). *M. officinalis* has also been recorded as a host of *E. pisi* in several countries, but not recorded for Taiwan (Braun and Cook 2012; Farr and Rossman 2024). Current studies, however, indicate *E. pisi* on *Melilotus* should be assigned to the *E. trifoliorum* complex by rDNA sequence analyses (Bradshaw et al. 2025a). These specimens, therefore, represent the first record of *Erysiphe trifoliorum* species complex on *M. officinalis* in Taiwan.

**Fig. 11:**
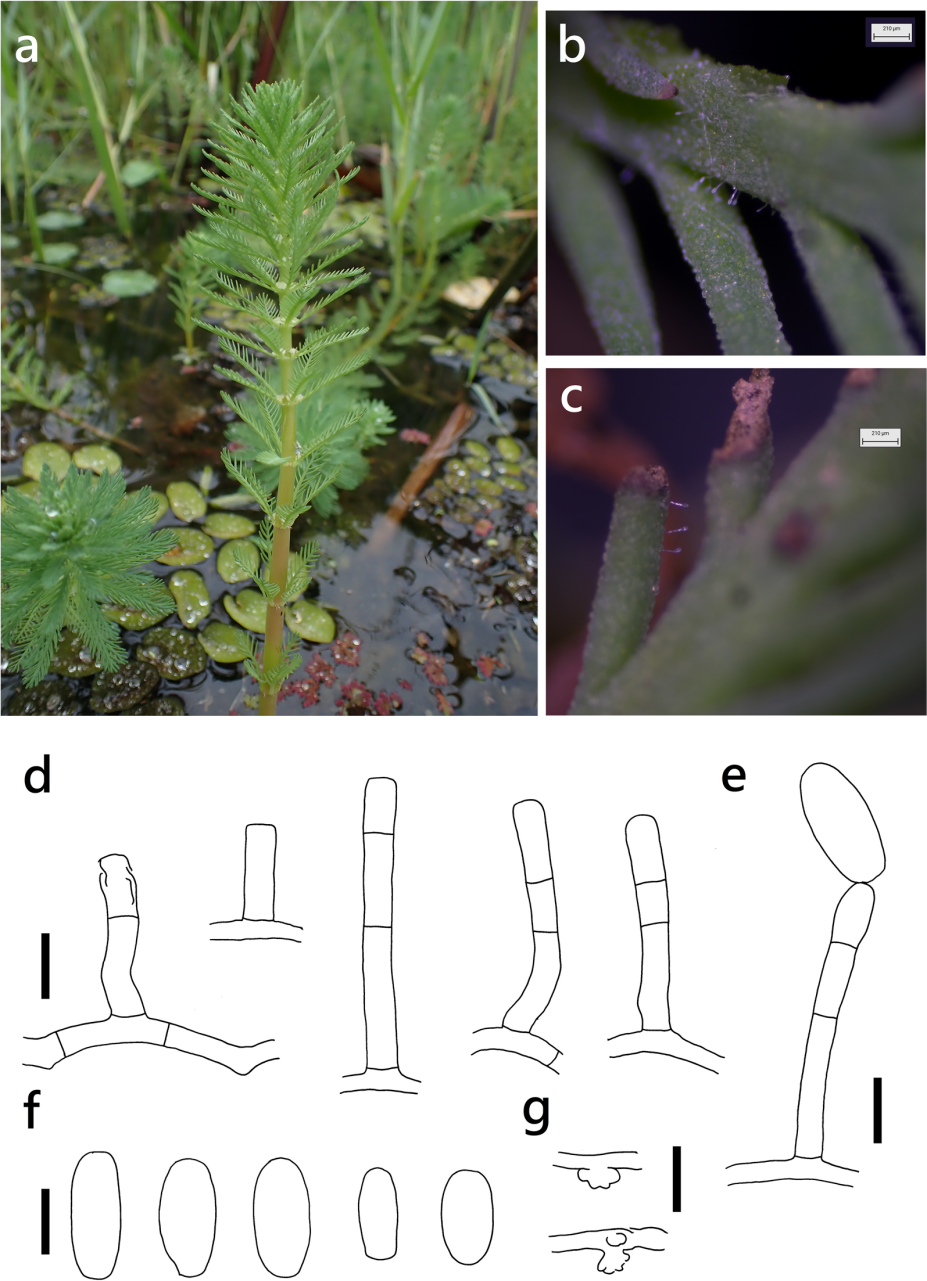
*Erysiphe trifoliorum* species complex on *Myriophyllum aquaticum* (R. Kirschner 5996). **a**. Habitat photo of *Myriophyllum aquaticum*. **b**-**c**. Magnified view of the infected leaf surface. **d**. Conidiophores with slightly curved foot cells. **e**. Conidiophore with a mature conidium at the terminal end. f. Conidia g. Appressoria. Scale bars d = 10 μm, all others = 20 μm

***Erysiphe trifoliorum ***species complex on *Myriophyllum aquaticum* (Haloragaceae) Figure 11

**Specimen** On *Myriophyllum aquaticum* (Vell.) Verdc., TAIWAN, Taipei City, Daan District, National Taiwan University, Liugong Canal, ca. 25.016291 N, 121.539843 E, ca. 10 m a.s.l., 8. Apr. 2024, R. Kirschner 5996 (TNM), ITS sequence in GenBank PV056466.

**Notes** The ITS sequence was 99% to 100% identical to published specimens labeled as *E. pisi* or *Erysiphe trifoliorum* in GenBank. Though *Myriophyllum aquaticum* is a popular plant in aquatic gardens, there are only two records of *M. aquaticum* infected by Erysiphaceae, which are recorded as *Microsphaera alphitoides* and *Oidium* sp. (Alfieri et al. 1984; Pennycook 1989; Farr and Rossman 2024). However, these two records are relatively old identifications based on traditional species concepts. Thus, this fungus was identified as *E. trifoliorum* species complex by the results of BLAST searches, which need further analysis to resolve the complex (Bradshaw et al. 2025a). This specimen is the first record of *Erysiphe trifoliorum* species complex on *Myriophyllum aquaticum* worldwide.

**Fig. 12:**
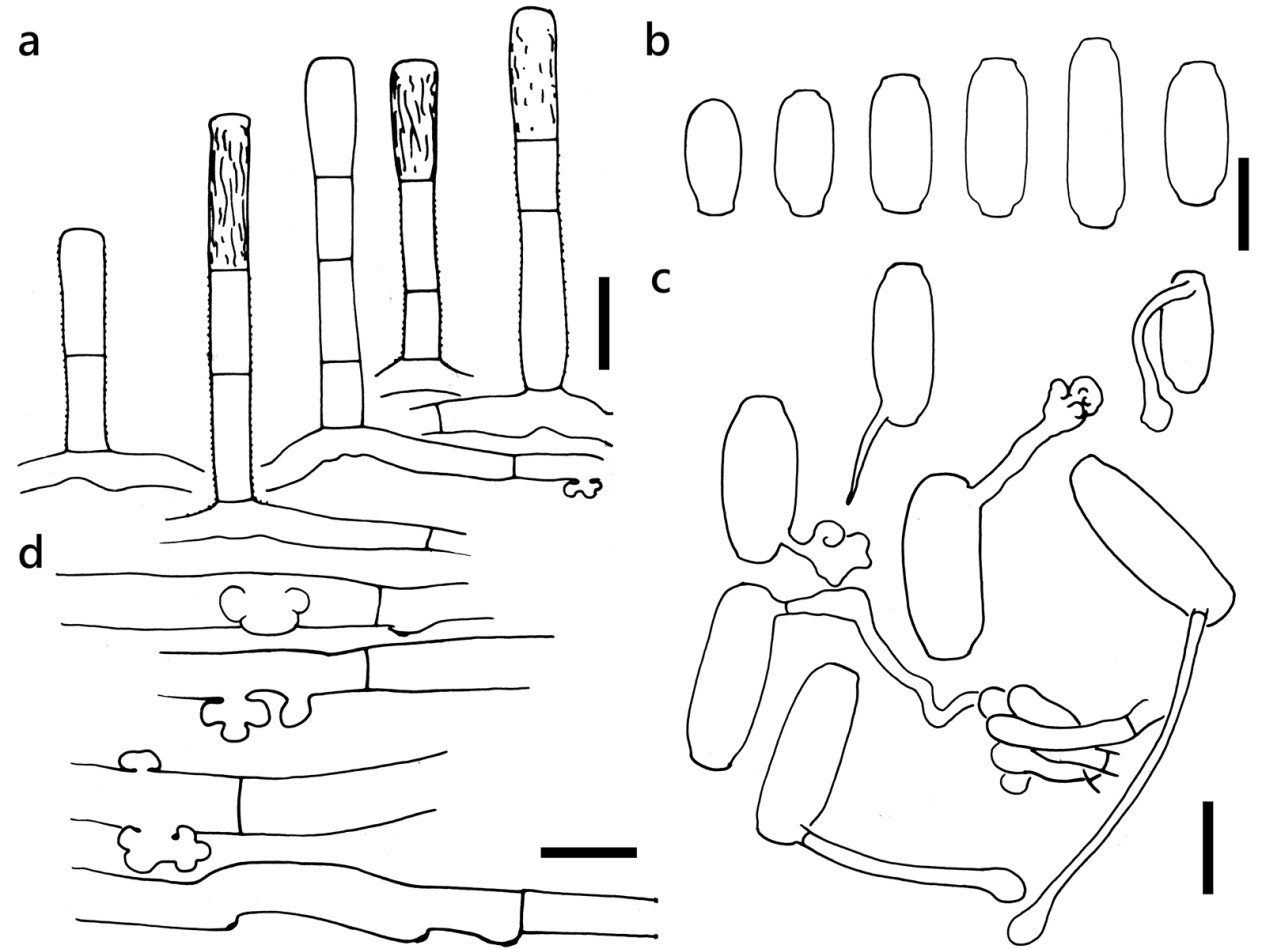
*Erysiphe sedi* on *Kalanchoe thyrsiflora* (R. Kirschner 4708). **a**. Conidiophores **b**. Conidia. **c**. Conidia with germination hyphae and appressoria **d**. Appressoria. Scale bars = 20 μm

***Erysiphe sedi*** U. Braun on diverse Crassulaceae Figure 12

Description (based on R. Kirschner 4708 on *Kalanchoe thyrsiflora*):

Colonies on initially green leaves which later forms purple spots with irregular margins, 1–4 mm diam. Hyphae smooth to verruculose, 5–8 μm wide. Hyphal appressoria mostly single, rarely in opposite pairs, nipple-shaped to multilobed. Conidiophores arising from about the middle of hyphal mother cell, verruculose except for the apical presumptive conidium with fine longitudinal striation on the cell wall, composed of two to four cells, (52–)71–91(–95) × (8–)8.5–9(–10) μm (n = 30). Foot cell straight, (14–)20–34(–40) × 7–9(–10) μm (n = 30). Conidia doliiform-cylindrical, with predominantly parallel, rarely convex lateral sides, smooth with few scattered minute cylindrical scales when fresh, when old becoming longitudinally striated, (25–)29–38(–44) × (12–)13–14.5(–15) μm (n = 30), germinating at one end either with short hypha terminating in a multilobed appressorium, or a longer hypha terminating in a swollen knob.

**Specimens** On *Hylotelephium telephium* (L.) H. Ohba (wild plant), GERMANY, Hessen, Darmstadt-Eberstadt, Schlossstrasse, Kernesbellen, 22. Aug. 2016, R. Kirschner 4332 (TNM), ITS sequence in GenBank PV422611; on *Kalanchoe blossfeldiana* Poelln. (potted plant), TAIWAN, Taipei City, Wenshan District, Muzha area, 22. Mar. 2020, AKII 0064, ITS sequence in GenBank PV090752; on *Kalanchoe delagoensis* Eckl. & Zeyh. (potted plant), TAIWAN, Hualien County, Hualien City, Haulien Train Station, ca. 23.993053 N, 121.601759 E, ca. 10 m a.s.l., 13. Mar. 2020, AKII 0060 (TNM), ITS sequence in GenBank PV056443; on *Kalanchoe laetivirens* Desc. (potted plant), TAIWAN, Hualien County, Hualien City, Hualien Train Station, ca. 23.993053 N, 121.601759 E, ca. 10 m a.s.l., 13. Mar. 2020, AKII 0059, ITS sequence in GenBank PV056472; TAIWAN, Taipei City, Daan District, Jianguo Holiday Flower Market, ca. 25.037937 N, 121.537888 E, ca. 10 m a.s.l., 21. Jun. 2022, AKII 0156, ITS sequence in GenBank PV056451; TAIWAN, Taoyuan City, Zhongli District, Zhong Ping Road, ca. 24.955969 N, 121.222250 E, 07. May 2022, R. Kirschner 5581 (TNM); on *Kalanchoe thyrsiflora* Harv. (potted plant), TAIWAN, New Taipei City, Yingge District, plant shop, 4. Apr. 2019, R. Kirschner 4708 (= AKII 0011) (TNM), ITS sequence in GenBank PV056444.

**Notes** When comparing BLAST matches exceeding 600 bp, the ITS sequence of *E. sedi* on *H. telephium* from Germany showed 99–100% identity with 0–3 different positions with that of *E. sedi* on *K. blossfeldiana* and *Orostachys japonica* from Korea (JX173288 and KT748731 Cho et al. 2012, 2016, respectively) as well as on *K. pinnata* from China (KR091961, Tang et al. 2016). *E. pileae* (see above) and the binomial *E. sedi* were published independently in the same year 1981, but *E. sedi* by Braun (1981) a few months earlier than that by Zheng & Chen (1981). Although belonging to the *E*. *aquilegiae* species complex, *E*. *sedi* on Crassulaceae hosts is separately named (Ramos et al. 2024). *Kalanchoe blossfeldiana, K. delagoensis*, *K. laetivirens*, and *K. thyrsiflora* are new hosts of this species complex worldwide.

**Fig. 13:**
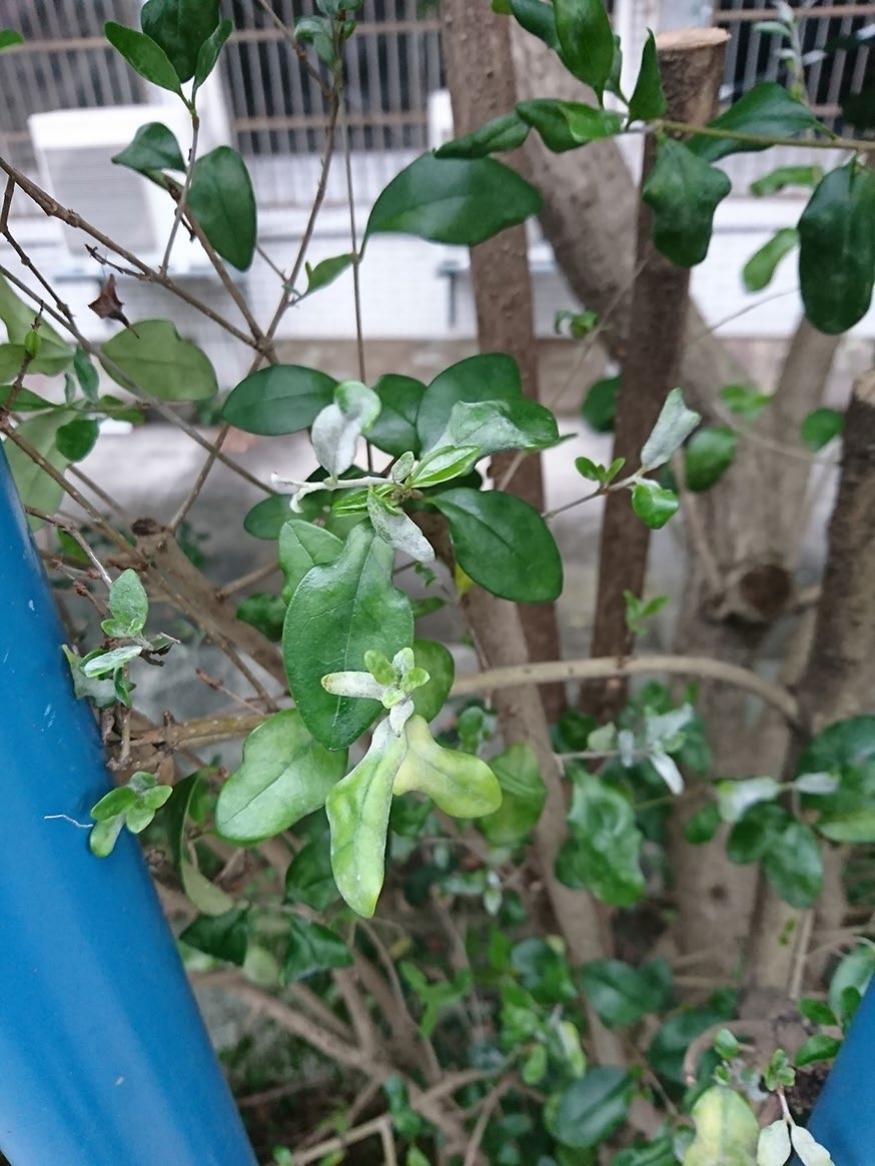
*Erysiphe syringae* on *Ligustrum liukiuense* (AKII 0172)

***Erysiphe syringae*** Schwein. on *Ligustrum liukiuense* (Oleaceae) Figure 13

**Specimens** On *Ligustrum liukiuense* Koidz., TAIWAN, New Taipei City, Yonghe District, Xiulang Elementary School, ca. 24.999445 N, 121.521329 E, ca. 10 m a.s.l., 13. May 2023, AKII 0172 (TNM), ITS sequence in GenBank PV056446.

**Notes** When ITS sequences exceeding 630 bp were compared, our specimen was 99% to 100% identical to ten published specimens labeled as *E. syringae* with 0 to 1 bp difference in GenBank, whereas the identity with other species was 97% or lower, with more than 15 different base pairs. *E. syringae* had been confused with *E. syringae-japonicae* due to overlapping host ranges, distribution, and morphological characteristics of the ascomata (Braun and Cook 2012). The phylogenetic status of *E. syringae* and *E. syringae-japonicae* was clarified by Seko et al. (2008, 2011), Takamatsu et al. (2016), and Bradshaw et al. (2024b, 2025a), confirming that *E. syringae* and *E. syringae-japonicae* are not closely allied based on rDNA analyses. Hitherto, there is no record of powdery mildews on *Ligustrum liukiuense* for Taiwan (Kuo 1998; Farr and Rossman 2024). This specimen is the first record of *E. syringae* on *Ligustrum liukiuense* in Taiwan.

**Fig. 14:**
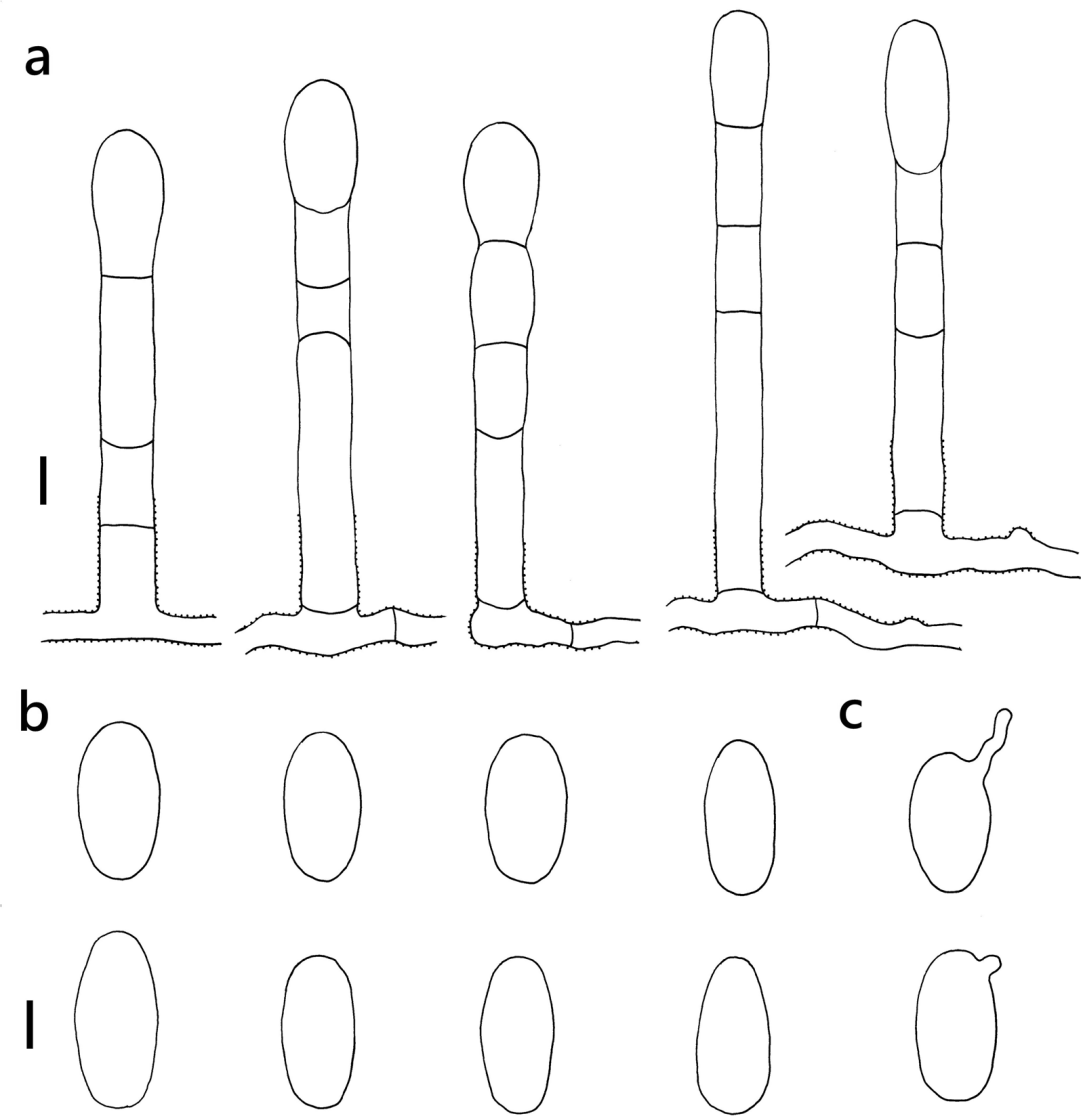
*Golovinomyces magnicellulatus* on *Nicotiana plumbaginifolia* (R. Kirschner 4922). **a**. Hyphae with lobed appressoria and conidiophores. **b**. Conidia. **c**. Conidia with germination hyphae. Scale bars = 10 μm

***Golovinomyces magnicellulatus*** (U. Braun) V.P. Heluta on *Nicotiana plumbaginifolia* (Solanaceae) Figure 14

**Description (based on RoKi 4922)** Mycelium amphigenous on leaves, forming irregular patches. Hyphae hyaline, smooth, 5–9 μm wide, with solitary, nipple-shaped appressoria. Conidiophores terminal on the surface of mother cells, mostly composed of 4 cells, (65–)75–104(–132) × 10–11(–13) μm (n = 30), foot cells straight, (35–)46–67(–77) × 10–11(–13) μm (n = 30), basal septum of the foot cell mostly at almost the same level as surface of supporting hypha. Conidia catenescent, ellipsoid-ovoid to doliiform, without fibrosin bodies, (28–)30–34(–38) × (15–)16–18(–19) μm (n = 30). Germ tubes subterminal or lateral.

**Specimens** On *Nicotiana plumbaginifolia* Viv., TAIWAN, Taipei City, Daan District, National Taiwan University, parking lot in front of the library, ca. 25.016916 N, 121.540128 E, ca. 10 m a.s.l., 28. Apr. 2020, R. Kirschner 4922 (TNM), ITS sequence in GenBank PV056465; Taipei City, Daan District, National Taiwan University, Student Activity Center, ca. 25.017705 N, 121.540116 E, ca. 10 m a.s.l., 10. May 2022, R. Kirschner 5583 (TNM), ITS sequence in GenBank PV056464.

**Notes** The ITS sequence was 99% to 100% identical to over twenty published specimens labeled as *Golovinomyces magnicellulatus* with 0 to 3 bp differences in GenBank, whereas the identity with other species was 96% or lower, with more than 26 bp differences. *Nicotiana* sp. was only recorded as the host of *G. orontii* and *G. longipes* (Braun and Cook 2012). Therefore, we treat *Nicotiana plumbaginifolia* as a new host of *G. magnicellulatus* worldwide. This also is a new fungus record for Taiwan.

**Fig. 15:**
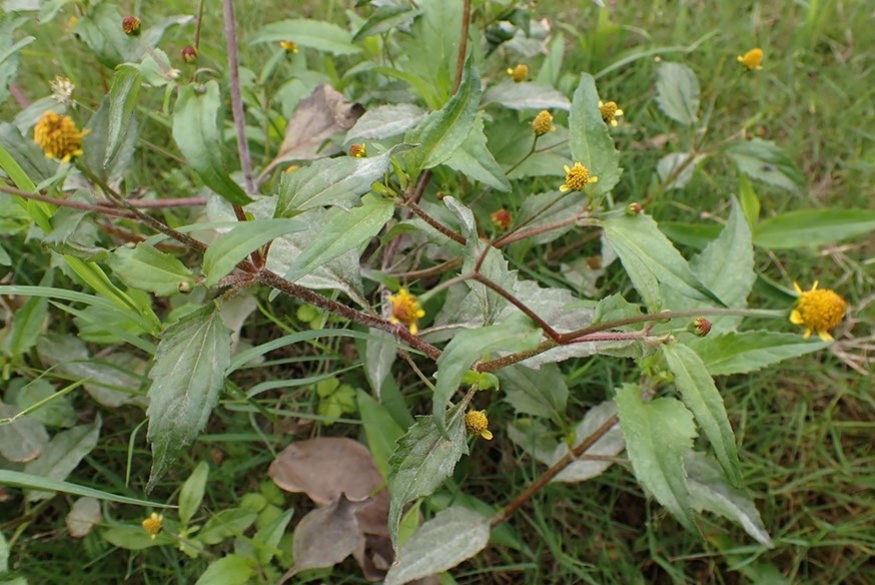
*Podosphaera xanthii* on *Acmella uliginosa* (R. Kirschner 5688)

***Podosphaera xanthii*** (Castagne) U. Braun & Shishkoff on *Acmella uliginosa* (Asteraceae) Figure 15

**Specimens** On *Acmella uliginosa* (Sw.) Cass., TAIWAN, New Taipei City, Zhonghe District, Xiulang Leisure Park, ca. 24.993086 N, 121.528480 E, ca. 5 m a.s.l., 30. Jan. 2023, R. Kirschner 5688 (TNM), ITS sequence in GenBank PV056463; Taipei City, Daan District, National Taiwan University, Liugong Irrigation Canal, ca. 25.018846 N, 121.537590 E, ca. 8 m a.s.l., 9. Apr. 2024, R. Kirschner 5999 (TNM), ITS sequence in GenBank PV056462.

**Notes** This fungus-host combination was recorded hitherto only for Thailand, with the host given as *Spilanthes iabadicensis* A.H. Moore (Meeboon and Takamatsu 2016). The host plant was identified to species with Chung et al. (2008). This specimen is the first record of *P. xanthii* on *Acmella uliginosa* in Taiwan.

**Fig. 16:**
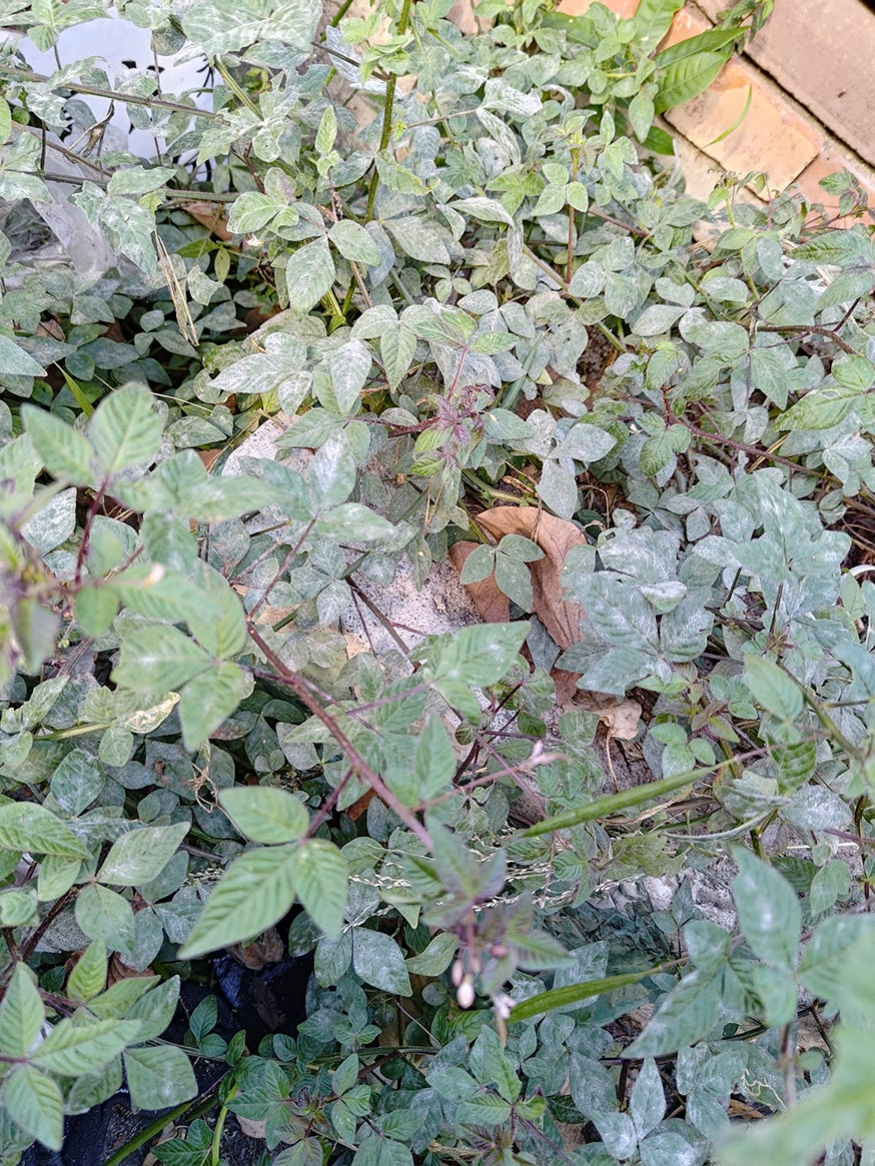
*Podosphaera xanthii* on *Cleome rutidosperma* (R. Kirschner 6079)

*Podosphaera xanthii* (Castagne) U. Braun & Shishkoff on *Cleome rutidosperma* (Cleomaceae) Figure 16

**Specimens** On leaves of *Cleome rutidosperma* DC., TAIWAN, Taipei City, Wenshan District, Zhinan Road Sec. 2, roadside opposite to main gate of Chengchi University, ca. 24.987550 N, 121.576736 E, ca. 15 m a.s.l., 8. Mar. 2020, R. Kirschner 4899 (TNM), ITS sequence in GenBank PV056456; Hsinchu City, Dongnan Str. 300, ca. 24.799584 N, 120.972957 E, ca. 10 m a.s.l., 01. Dec. 2024, R. Kirschner 6079 (TNM), ITS sequence in GenBank PV056461.

**Notes** On this host plant species, *P. xanthii* is the single known powdery mildew and has hitherto only been recorded for Thailand (Meeboon et al. 2016b). This specimen is the first record of *P. xanthii* on *Cleome rutidosperma* in Taiwan.

***Podosphaera xanthii ***(Castagne) U. Braun & Shishkoff on *Clinopodium gracile* (Lamiaceae).

**Specimens** On *Clinopodium gracile* (Benth.) Kuntze, TAIWAN, Nantou County, Lugu Township, National Taiwan University Experimental Forest, Fenghuang Nature Education Area, ca. 23.728710 N, 120.787910 E, ca. 860 m a.s.l., 8. May 2021, AKII 0133 (TNM); Nantou County, Lugu Township, National Taiwan University Experimental Forest, Xitou Nature Education Area, ca. 23.674304 N, 120.797547 E, ca. 1100 m a.s.l., 13. Feb. 2023, AKII 0164 (TNM), ITS sequence in GenBank PV056450.

**Notes** This fungus-host combination has previously been reported from Japan and Korea under the names *Sphaerotheca fuliginea* and *S. fusca*, respectively (Farr and Rossman 2024). However, these names were historically used in a broad sense. In current taxonomy, their usage has been narrowed and is now restricted to powdery mildews on *Doronicum* and *Veronica*, respectively (Braun and Cook 2012). Therefore, the powdery mildew found on *Clinopodium gracile* in this study is best referred to as *P. xanthii*, and this represents the first record of this species on this host in Taiwan.

**Fig. 17:**
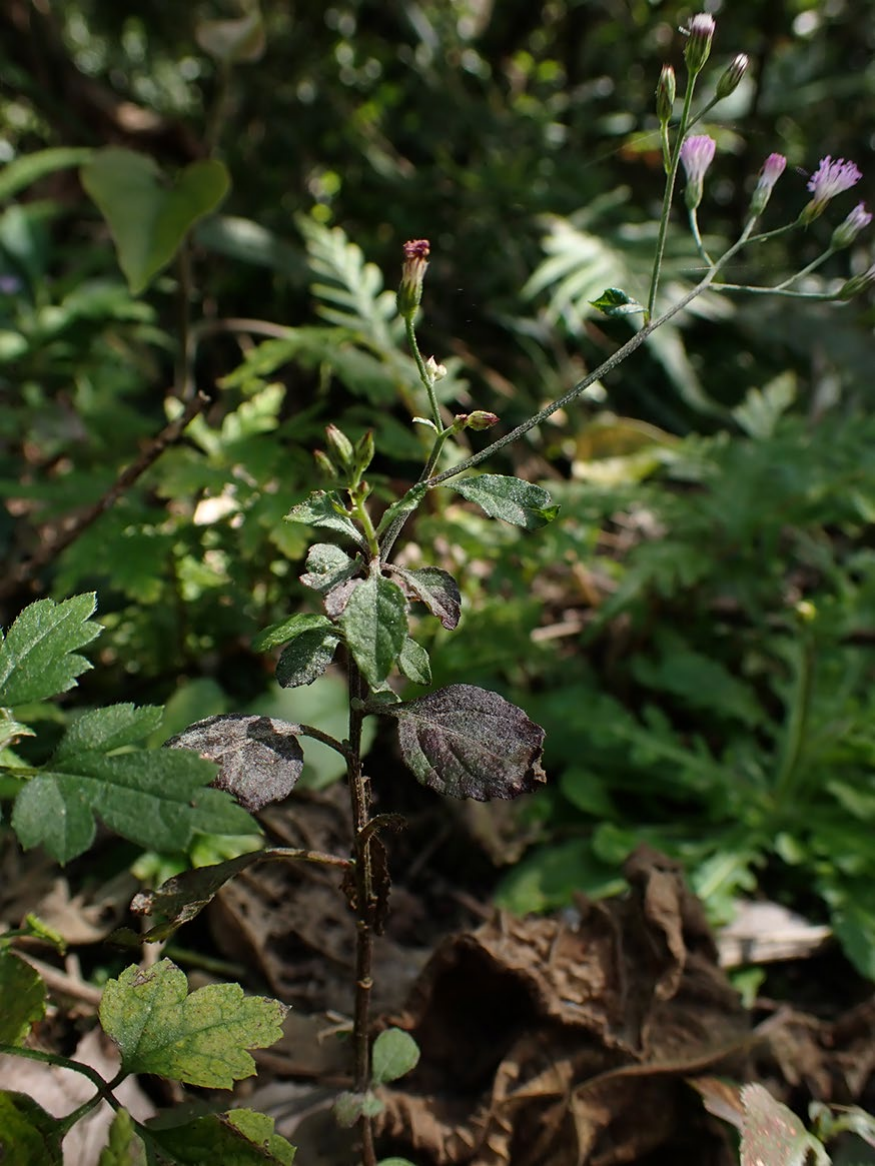
*Podosphaera xanthii* on *Cyanthillium cinereum* (R. Kirschner 5121)

***Podosphaera xanthii*** (Castagne) U. Braun & Shishkoff on *Cyanthillium cinereum* (Asteraceae) Figure 17

**Specimen** On leaves of small individuals of *Cyanthillium cinereum* (L.) H. Rob. [= *Vernonia cinerea* (L.) Less.], TAIWAN, Taipei City, Wenshan District, National Chengchi University, entry to Flying Dragon Hiking Trail, ca. 24.980181 N, 121.576512 E, ca. 115 m a.s.l., 16. Feb. 2021, R. Kirschner 5121 (TNM), ITS sequence in GenBank PV056455.

**Notes** The fungus was found only on rather poorly developed individuals of the host plant. Although *C. cinereum* is a common and widespread weed, infection by powdery mildews is very rarely encountered. On this host plant species, *P. xanthii* has hitherto only been recorded for Thailand (Meeboon et al. 2016b). This specimen is the first record of *P. xanthii* on *C. cinereum* in Taiwan.

**Fig. 18:**
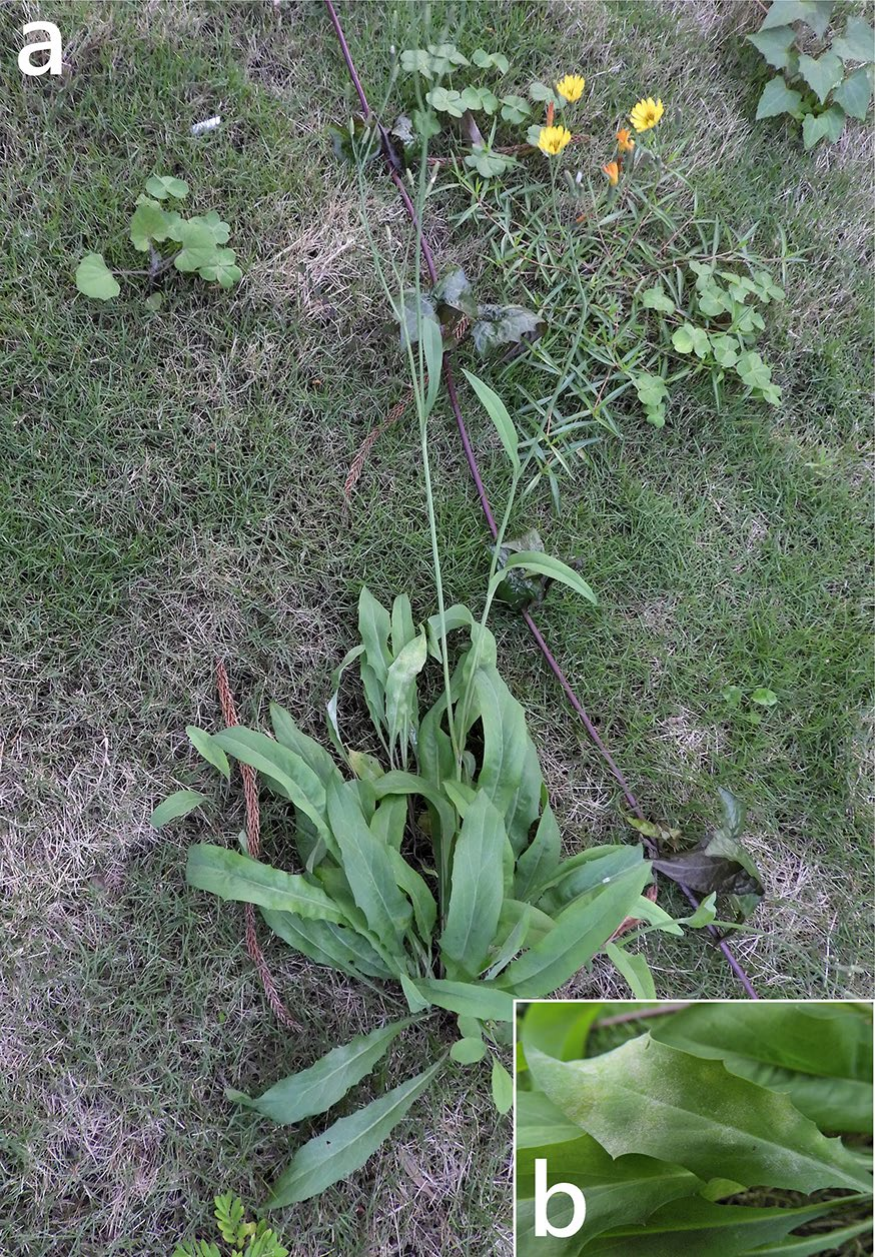
*Podosphaera xanthii* on *Ixeris chinensis* (R. Kirschner 4867). **a**. Habit of host plant. **b**. Magnified view of the infected leaf surface.

***Podosphaera xanthii*** (Castagne) U. Braun & Shishkoff on *Ixeris chinensis* (Asteraceae) Figure 18

**Description** Mycelium amphigenous on leaves and petioles, forming irregular patches. Hyphae hyaline, walls thin, smooth, 4–8 μm wide, with solitary, nipple-shaped appressoria. Conidiophores terminal on the surface of mother cells, composed of 4–7 cells, mostly 5-celled, (57–)66–109(–150) × 9–11(–12) μm (n = 30), foot cells curved at the base, constricted at basal septum, (31–)40–63(–81) × 9–11(–12) μm (n = 30), basal septum of the foot cell mostly at almost the same level as surface of supporting hypha. Conidia catenate, ellipsoid-ovoid to doliiform, with distinct fibrosin bodies, (26–)29–32(–34) × 15–17(–18) μm (n = 30). Germination not found.

**Specimen** On leaves of *Ixeris chinensis* (Thunb.) Kitag., TAIWAN, Taipei City, Daan District, Keelung Road close to National Taiwan University, ca. 25.015830 N, 121.542391 E, ca. 10 m a.s.l., 14. Jan. 2020, R. Kirschner 4867 (TNM), ITS sequence in GenBank PV056458.

**Notes** According to Braun & Cook (2012) and USDA fungal databases (Farr and Rossman 2024), *I. chinensis* has hitherto not been recorded as host of *P. xanthii*. We, therefore, treat *I. chinensis* as a new host of *P. xanthii*.

**Fig. 19:**
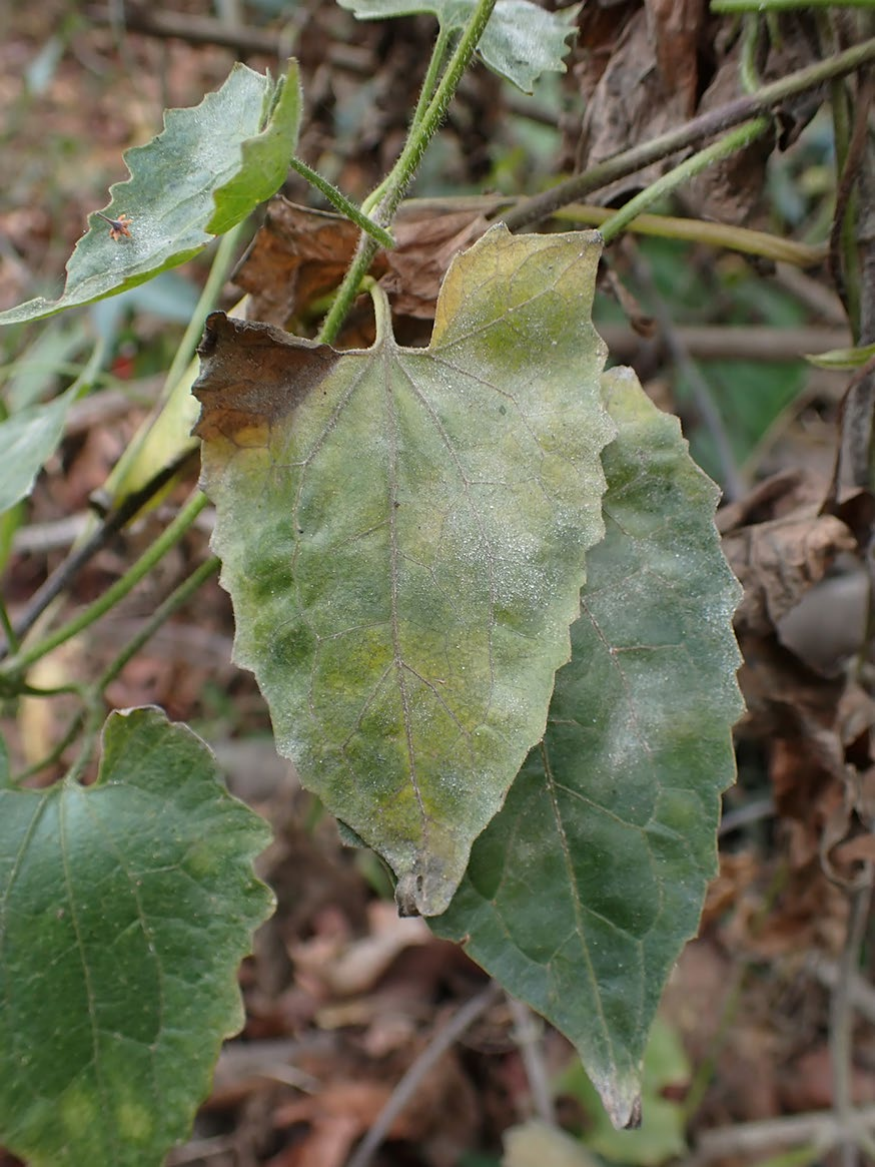
*Podosphaera xanthii* on *Mikania micrantha* (R. Kirschner 6002)

***Podosphaera xanthii*** (Castagne) U. Braun & Shishkoff on *Mikania micrantha* (Asteraceae) Figure 19

**Specimen** On leaves and stems of *Mikania micrantha* Kunth [= *Archibaccharis serratifolia* (Kunth) S.F. Blake], TAIWAN, Nantou County, Shuili Township, Shuixin Road Sec. 1, No. 547, ca. 23.805221 N, 120.860351 E, ca. 300 m a.s.l., 13. Apr. 2024, R. Kirschner 6002 (TNM), ITS sequence in GenBank PV056457.

**Notes** In our specimen, we found several “giant” cylindrical conidia up to almost 60 µm long. The leaves were also infested by a mite. *Mikania micrantha* is one of the most noxious invasive weeds in Taiwan. An intentionally introduced foreign rust fungus as well as accidentally introduced fungi such as *Cercospora mikaniicola* F. Stevens have been without significant effect (Kirschner 2015b). Even in tropical America, the native range of the plant, no powdery mildew has been recorded for this host (Barreto & Evans 1995). We, therefore, consider this specimen to be the first record of *Podosphaera xanthii* on *Mikania micrantha* worldwide. As *M. micrantha* prevails as a strongly invasive weed, the singular discovery of the powdery mildew does not suggest this species to be an efficient fungal agent for biological control.

**Fig. 20:**
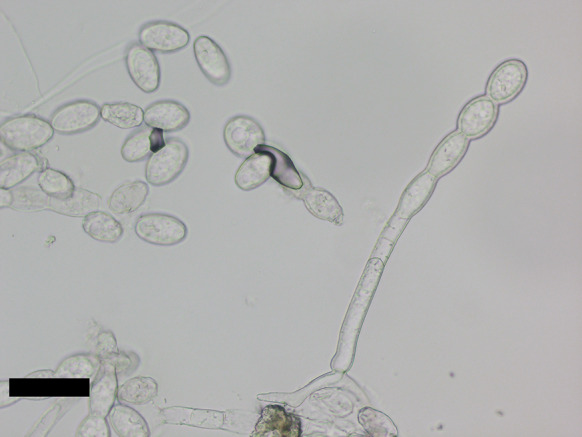
Conidiophore and conidia of *Podosphaera xanthii* (AKII 0195). Scale bar = 50 μm

***Podosphaera xanthii*** (Castagne) U. Braun & Shishkoff on *Parthenium hysterophorus* (Asteraceae) Figure 20

**Specimen** On leaves of *Parthenium hysterophorus* L., TAIWAN, Kaohsiung, Gangshan, roadside between Gangshan train station and MRT South Gangshan station, 24. Feb. 2024, AKII 0195 (TNM), ITS sequence in GenBank PV056452.

**Notes** Hitherto, several further powdery mildew species have been occasionally recorded on this host, namely *Golovinomyces cichoracearum* and *G*. *spadiceus* (Braun and Cook 2012; Farr and Rossman 2024). However, there is no record of Erysiphaceae species on this host in Taiwan. This specimen represents the first record of *Podosphaera xanthii* on *Parthenium hysterophorus* worldwide. The host, a poisonous and invasive plant species, was introduced from Latin America to Taiwan and was first recorded in 1986 (Chang-Yang et al. 2022). Powdery mildews might be potential biocontrol agents for the host.

***Podosphaera xanthii*** (Castagne) U. Braun & Shishkoff on *Scoparia dulcis* (Plantaginaceae).

**Specimen** On leaves, calyces, and stems of *Scoparia dulcis* L., TAIWAN, New Taipei City, Xindian District, City Highway near Ciji Hospital, ca. 24.987763 N, 121.535451 E, ca. 12 m a.s.l., 7. Apr. 2024, R. Kirschner 5998 (TNM), ITS sequence in GenBank PV056454.

**Notes** Up to now, only *Oidium scopariae* (N.D. Sharma & A.C. Jain) Bhagyan. & Ramachar and *Oidium* sp. have been recorded on this host species from India, USA, and Venezuela (Farr & Rossman 2024). The catenate conidia mentioned in the original description (Sharma & Jain 1975) suggest a species of *Golovinomyces* or *Podosphaera*. Braun & Cook (2012) listed *O. scopariae* as synonym of *Golovinomyces orontii* (Castagne) V.P. Heluta as well as in the list of excluded and doubtful species. We found fibrosin bodies in the conidia of our specimen and molecular sequence data confirmed the identification as *P. xanthii*. Our data indicate that *O. scopariae* may be a synonym of *P. xanthii* rather than of *G. orontii*, but *S. dulcis* may be confirmed also as host of *G. orontii*. Since the potential synonymy cannot yet be clarified, the taxonomic situation is quite different from previous identifications of *P. xanthii* as *Podosphaera/Sphaerotheca fuliginea/fusca*, where according to Braun & Cook (2012) there is no doubt that those previous identifications indicated *P. xanthii*. Therefore, we treat *S. dulcis* as a new host of *P. xanthii*.Reference: Reference [Chen (2003b), Fu et al. (2004), Hsieh (1983a), Hsieh (1983b), Hsieh (1983c), Hsieh (1986), Kuo et al. (1989), Yen and Wang (1973)] was provided in the reference list; however, this was not mentioned or cited in the manuscript. As a rule, if a citation is present in the text, then it should be present in the list. Please provide the location of where to insert the reference citation in the main body text.new citations of these references inserted into the Introduction section, except for Chen (2003b) which is deleted. Therefore Chen (2003a) is changed to Chen (2003) in the citation and reference.

**Fig. 21:**
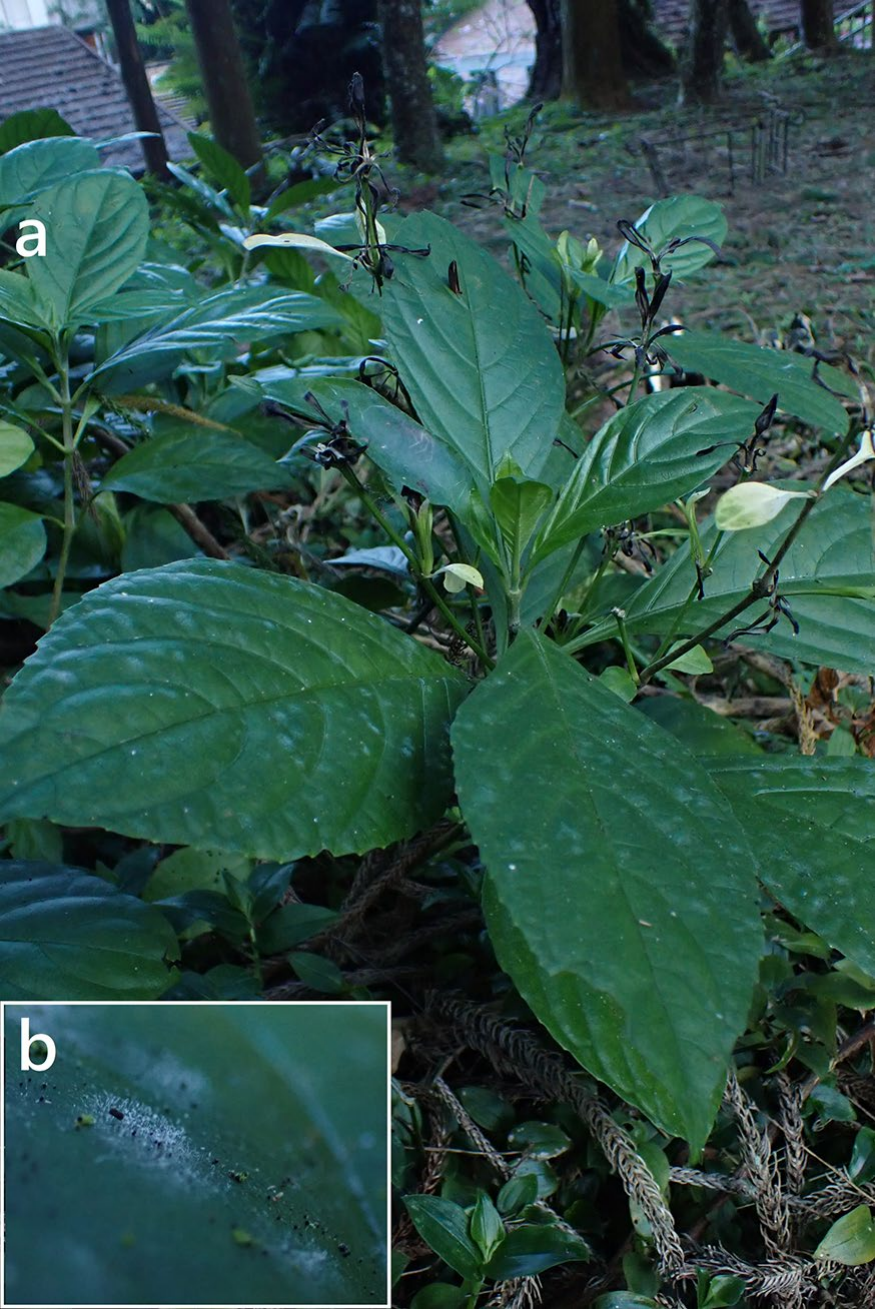
*Podosphaera xanthii* on *Strobilanthes cusia* (AKII 0165). **a**. Habit of host plant with whitish leaf spots. **b**. Magnified view of the infected leaf surface

***Podosphaera xanthii*** (Castagne) U. Braun & Shishkoff on *Strobilanthes cusia* (Acanthaceae) Figure 21

**Specimen** On leaves of *Strobilanthes cusia* (Nees) Kuntze, TAIWAN, Nantou County, Lugu Township, National Taiwan University Experimental Forest, Xitou Nature Education Area, ca. 23.674304 N, 120.797547 E, ca. 1100 m a.s.l., 13. Feb. 2023, AKII 0165 (TNM), ITS sequence in GenBank PV056449.

**Notes** According to Braun & Cook (2012) and USDA fungal databases (Farr and Rossman 2024), *Strobilanthes cusia* has hitherto not been recorded as host of *P. xanthii*. We, therefore, treat *Strobilanthes cusia* as a new host of *P. xanthii*. The host plant was economically important for blue stain of clothes in the past, which is a niche market in Taiwan.

**Fig. 22:**
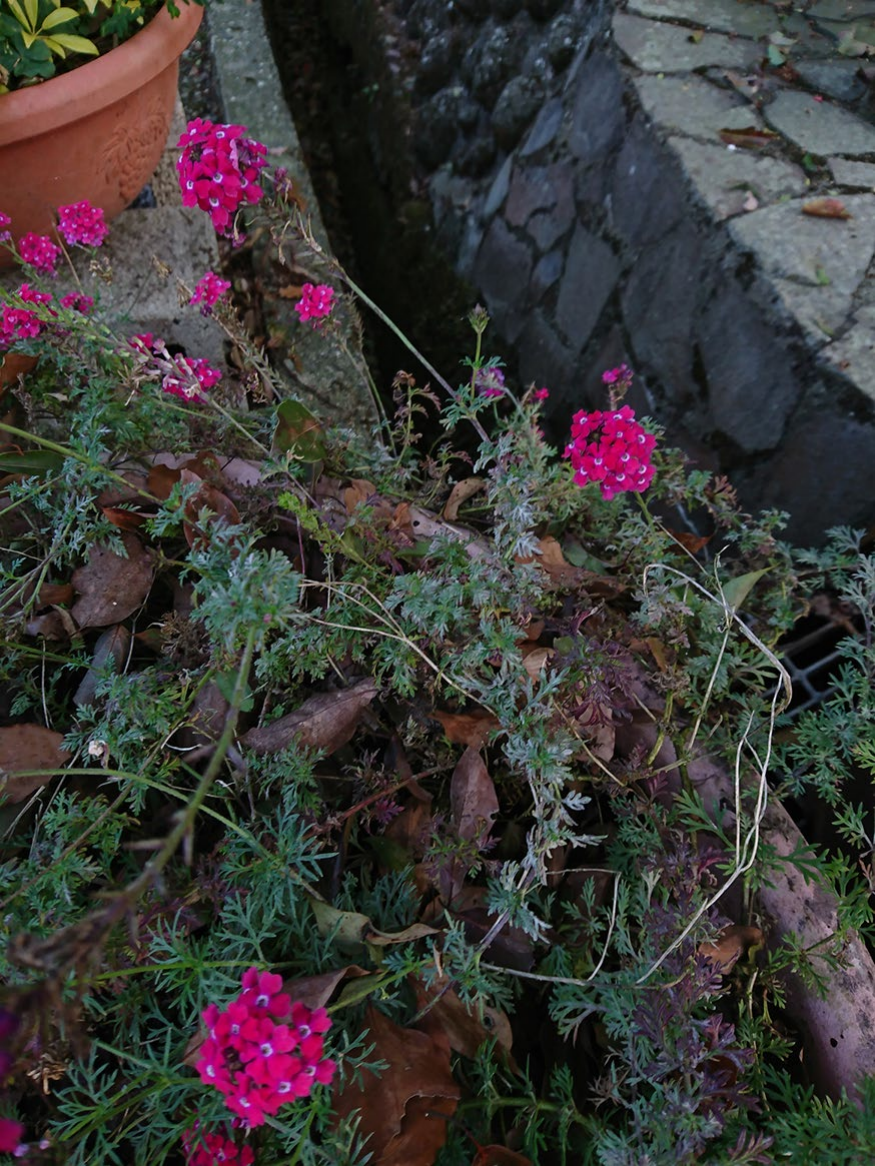
*Podosphaera xanthii* on *Verbena* × *hybrida* (R. Kirschner 5985)

***Podosphaera xanthii*** (Castagne) U. Braun & Shishkoff on *Verbena* × *hybrida* (Verbenaceae) Figure 22

**Specimens** On leaves and stems of *Verbena* × *hybrida* Groenl. & Rümpler [= *Glandularia* × *hybrida* (hort. ex Groenl. & Rümpler) G.L.Nesom & Pruski], TAIWAN, Nantou County, Lugu Township, National Taiwan University Experimental Forest, Fenghuang Nature Education Center, ca. 23.729953 N, 120.787896 E, ca. 800 m a.s.l., 22. & 29. Apr. 2024, R. Kirschner 5985 (TNM), ITS sequence in GenBank PV056460; On leaves of *Verbena* sp., TAIWAN, Nantou County, Lugu Township, National Taiwan University Experimental Forest, Fenghuang Nature Education Area, ca. 23.728710 N, 120.787910 E, ca. 860 m a.s.l., 19. Mar. 2023, AKII 0124 (TNM), ITS sequence in GenBank PV056440.

**Notes** The hybrid formula of this artificial cross is *V. peruviana* × *V. phlogiflora* × *V. platensis* × *V. tweedieana* (Plants of the World Online von Board of Trustees of the Royal Botanic Gardens, Kew). Several countries have been recorded for *P. xanthii* on *Glandularia* (*Verbena*) species and hybrids, but Taiwan is not included (Farr and Rossman 2024). *Verbena* sp., therefore, is a new host record of *P. xanthii* in Taiwan.

## Discussion

### Numbers of Erysiphaceae species and their hosts in Taiwan

**Fig. 23:**
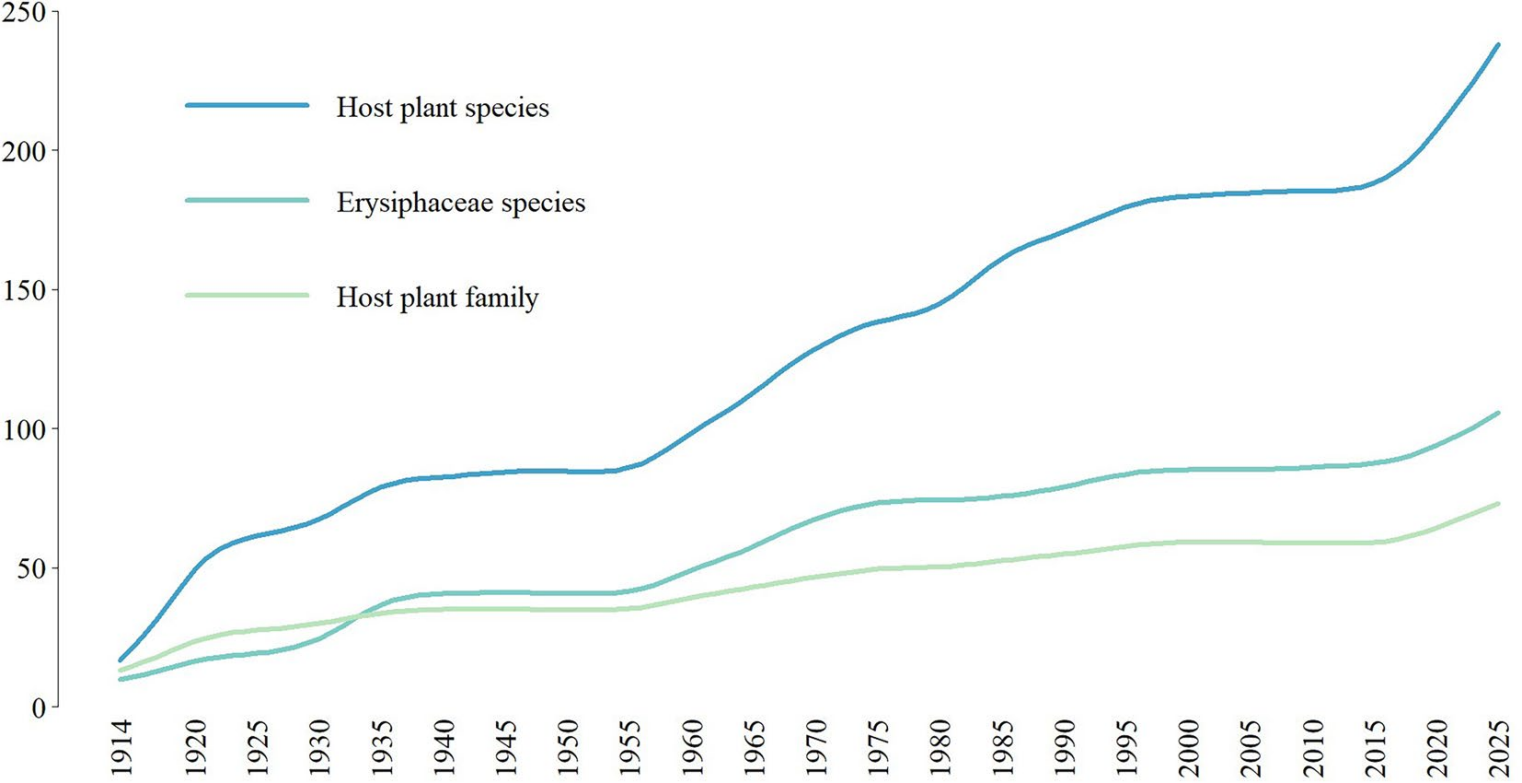
Cumulative locally estimated scatterplot smoothing curve of Erysiphaceae species and their host and host families in Taiwan (1914–2025). Span = 0.5

According to the updated checklist, the numbers of powdery mildew species, host species, and host families known for Taiwan are 109, 246, and 75, respectively (Figure 23). This includes 29 records from this study and excludes doubtful records due to lack of evidence or incomplete identification. The numbers of species of Erysiphaceae represents approximately 10% of the species number of Erysiphaceae known worldwide. When analyzing these numbers, we were surprised to find that the ratio between powdery mildew species, host species, and host families has remained relatively stable since 1933. The ratio of Erysiphaceae species to host species was ca. 1:2 in the 1930 s and has gradually increased to 1:2.25, most likely due to the discovery that some powdery mildew species have a broader host range covering a few to many host families, e.g., *Podosphaera xanthii* and *Erysiphe quercicola* (Braun and Cook 2012). The ratio of Erysiphaceae species to host families was 1:0.89 in the 1930 s and has gradually decreased to 1:0.68, which indicates that more than one powdery mildew species can occur on the same host family, e.g., papaya (Caricaceae) has been recorded as host of several Erysiphaceae species (Braun et al. 2017). According to the updated checklist, the top three plant families with the largest number of fungus-host combinations are Asteraceae (41 fungus-host combinations), Fabaceae (30 fungus-host combinations), and Rosaceae (20 fungus-host combinations), which exactly reflect the rank of species count in plant families. The top families by species count in Taiwan flora are Orchidaceae, Poaceae, Asteraceae, Fabaceae, Cyperaceae, and Rosaceae (Hsieh 2002). Among them, Orchidaceae and Cyperaceae are not recorded as hosts of Erysiphaceae, and Poaceae are primarily infected by *Blumeria* spp. In the past 30 years, the number of Erysiphaceae species known for Taiwan has increased by ca. 24 species (from 85 to 109), while the number of host plant species has increased by around 63 species (from 182 to 245). This suggests that the present knowledge gap lies not in new taxonomic records of species but in host plant spectra.

The cumulative curve of Erysiphaceae species recorded for Taiwan reflects the historical events and human activities of the time. For example, the cumulative curve flattens during two periods: 1930–1950, reflecting the Second Sino-Japanese War, and 1990–2010, corresponding to Taiwanese agricultural policies at the time that mainly focused on controlling plant diseases, not on investigating the taxonomy of those pathogens (Liaw 2001; Chen 2014).

### Update of names of host plants and fungi

While plant nomenclature tends to be more stable than that of fungi, some names have changed over the past century since the first report of powdery mildews in Taiwan (Sawada 1914; Yeh et al. 2020). Updating host plant names is very important, as synonyms can cause confusion to mycologists, e.g., Meeboon et al. (2016) reported the same host record twice under its synonyms, *Chamaesyce hirta* (L.) Millsp. and *Euphorbia hirta* L.

In contrast, fungal nomenclature and fungal species concepts are complicated and variable, e.g., the dual nomenclature which was applied for over one century was replaced by One-Fungus = One-Name (1F =  1 N) nomenclature in 2012. Failing to update the names of host plants and fungi may result in false new records. One example is that Xiao et al. (2021) reported a new host record of *Podosphaera fusca* on *Vigna unguiculata*. In Kuo’s list (1998), this host was listed as *Vigna sinensis* (a synonym of *V. unguiculata*) and the associated fungus was recorded as *Sphaerotheca fuliginea*, a name that was historically used in a broad sense for what is now treated as *P. xanthii* (Braun and Cook 2012). Another example is *P*. *xanthii* on *V*. *radiata* reported as a new host record for Taiwan by Sheu et al. (2021), which had been recorded in Kuo’s list (1998) with the fungus as *S. fuliginea* and updated as *P*. *xanthii* in Taiwan (Yeh et al. 2020).

### Challenges in verifying historical records under changing standards

The fungus-host combinations of Erysiphaceae in Taiwan now comprise ca. 308 records (including this study). Over two-thirds of these records (228 records) were published before 2010, which were based on the traditional identification concepts, except for one record by Chen et al. (2008). Among these records, some of them are doubtful since the fungal species have revealed to represent species complexes including species which cannot be identified without molecular analyses, e.g., *Erysiphe alphitoides*, *Erysiphe aquilegiae*, and *Phyllactinia guttata*. In the *E*. *aquilegiae* species complex we follow the tentative separate species concepts based on the assumed host specificities (Bradshaw et al 2024b, a). Furthermore, species concepts of Erysiphaceae have changed and some species are considered to have a broader host range than before, e.g., *Podosphaera xanthii* and *Erysiphe quercicola* (Braun and Cook 2012)*.* Due to a high ratio of species splitting or cryptic species, species of *Golovinomyces* and *Blumeria* are not resolvable by ITS sequences only, they need additional molecular data for clarification. Thus, the re-investigation of these old records is important but difficult to accomplish. One challenge is that extracting genomic DNA from dried specimens of Erysiphaceae is difficult, largely depending on the preservation condition of the specimens. Some specimens contain few fungal tissues and have been deposited for a long time, resulting in little to no remaining DNA.

### Challenges in broadening the scope of Erysiphaceae in Taiwan

With globalization accelerating, more plant species are being introduced to Taiwan every year. As a result, fungi are introduced alongside plants (Yeh et al. 2018). In addition, new plant species continue to be described for Taiwan (Lu et al. 2019; Wang et al. 2020, 2024; Huang et al. 2024), and the number of naturalized foreign plant species is increasing annually (Chang-Yang et al. 2022), simultaneously expanding the range of potential hosts for pathogenic fungi. These fungi may be native to Taiwan or naturalized there a long time ago. This is, however, difficult to demonstrate due to the lack of data on the geographic distribution of fungi (Kirschner 2015d).

The estimated number of species of Erysiphaceae existing worldwide is 10,000 (Rossman 1994; Hawksworth 2001). However, there are hitherto only around 900 species of Erysiphaceae described (Shirouzu et al. 2020; Bradshaw et al. 2024a), which is quite far from the estimation. The numbers mentioned above reveal that continuous support for biodiversity studies is imperative.

## Conclusions

The checklist of Erysiphaceae known for Taiwan according to data in literature has been compiled and updated as well as expanded by 29 new host records of powdery mildews based on specimens investigated in the context of the present study. The numerous new host records of powdery mildews indicate that our knowledge on host ranges is incomplete. Data are insufficient to assess host range extension from introduced to native hosts and vice versa. The old doubtful records identified with outdated species concepts and the lack of DNA data show that our knowledge of Erysiphaceae is far from complete. Powdery mildews as obligate plant pathogenic fungi forming conspicuous symptoms are supposed to belong to the best candidates for studying the globalization of pathogens (Glawe 2008). This is, however, apparently challenging due to limitations concerning numbers of researchers and funding. To break through these limitations, continuous attention and support for investigation on biodiversity are necessary.

## Data Availability

Deposit of data and materials is given in the Methods Section.
